# Biochemical characterization of two *Brassica oleracea* nitrile-specifier proteins

**DOI:** 10.3389/fpls.2026.1740844

**Published:** 2026-01-29

**Authors:** Kudzai Gracious Mbudu, Katja Witzel, Ute Wittstock, Frederik Börnke, Franziska Sabine Hanschen

**Affiliations:** 1 Plant Quality and Food Security, Leibniz Institute of Vegetable and Ornamental Crops, Grossbeeren, Germany; 2 Institute of Biochemistry and Biology, University of Potsdam, Potsdam, Germany; 3 Science Support Platform, Leibniz Institute of Vegetable and Ornamental Crops, Grossbeeren, Germany; 4 Institute of Pharmaceutical Biology, Technische Universität Braunschweig, Braunschweig, Germany; 5 Bavarian Health and Food Safety Authority (LGL), Erlangen, Germany; 6 Institute of Food Technology and Food Chemistry, Technische Universität Berlin, Berlin, Germany

**Keywords:** biochemical characterization, *Brassica oleracea* (cabbage), gene family, isothiocyanate, nitrile, nitrile-specifier proteins, phylogenetic analysis, protein domain evolution

## Abstract

*Brassica oleracea* vegetables (e. g. cabbages) form bioactive isothiocyanates (ITCs) from glucosinolate (GLS) hydrolysis. However, enzymatic activity, acidic pH (below pH 5), and ferrous ions (Fe^2+^) can promote nitrile release, reducing the ITC amount. In *Arabidopsis thaliana*, nitrile-specifier proteins (NSPs) promote nitrile formation upon GLS hydrolysis. Here, we report the functional characterization of two *Brassica* NSPs from *B. oleracea* and the *in silico* identification of candidate genes encoding a family of sixteen *B. oleracea* NSPs closely related to the *A. thaliana* NSPs and the likely ancestral protein, XP_013585314.1. High conservation of the iron-binding triad (EXXXDXXXH), characteristic of specifier proteins, was confirmed in the putative BoNSPs. Biochemical characterization of two *B. oleracea* NSP isoforms, BoNSP2 (XP_013609641.1) and BoNSP11 (XP_013587057.1), revealed increased NSP activity in the presence of added Fe^2+^. Both BoNSP isoforms affected hydrolysis of five GLS differently *in vitro*, suggesting differential substrate specificity. BoNSP2 showed higher nitrile formation from indol-3-ylmethyl GLS than from 4-(methylsulfinyl)butyl GLS. In contrast, BoNSP11 similarly increased nitrile formation from indol-3-ylmethyl GLS, three aliphatic GLS and benzyl GLS. BoNSP2 and BoNSP11 were most active between pH 7 and pH 8. This study identifies and characterizes the first NSPs in *B. oleracea* vegetables at the molecular level.

## 1 Introduction

*Brassica oleracea* vegetables, which include crops such as cabbage, kale and cauliflower, hold significant economic importance and are cultivated on a global scale (Zhang et al., 2024). Moreover, *B. oleracea* vegetables are a rich source of various phytochemicals, including phenolic compounds, carotenoids and glucosinolates (GLSs) (Kaulmann et al., 2014; Statilko et al., 2024). GLSs play a role in plant chemical defense against generalist and specialist herbivores as well as microbial pathogens, enhance abiotic stress tolerance, and their consumption is linked to numerous human health-beneficial properties (Connolly et al., 2021; Jeschke et al., 2017; Nicolas-Espinosa et al., 2023).

GLSs are nitrogen and sulfur-containing plant secondary metabolites in *Brassicaceae* and related families. They are synthesized from amino acids. Their structure is characterized by a (*Z*)-*N*-hydroximinosulfate ester core linked to a β-d-glucose via a thioglucosidic bond and to a variable side chain (**Supplementary Figure S1**). Based on the precursor amino acid, GLSs are categorized as aliphatic, benzenic or indolic (Blažević et al., 2020). They are substrates of endogenous β-thioglucosidases known as myrosinases (EC 3.2.1.147). When myrosinases come into contact with GLSs, for example, upon tissue damage, they hydrolyze GLSs leading to the formation of volatile and bioactive products, including isothiocyanates (ITCs), nitriles (cyanides; CNs), epithionitriles (ETNs) and organic thiocyanates (Burow et al., 2007; Kuchernig et al., 2011; Wittstock and Burow, 2010) (**Supplementary Figure S1**). Thus, bioactivity is mostly due to the hydrolysis products and not the GLS itself (Wittstock and Burow, 2010). ITCs are spontaneously formed upon GLS hydrolysis through a Lossen-like rearrangement, where the side chain group transfers from the oxime carbon to the adjacent nitrogen atom (**Supplementary Figure S1**) (Halkier and Gershenzon, 2006). ITCs have numerous human health-beneficial properties when consumed through GLS-containing vegetables (Connolly et al., 2021). Moreover, among the wide array of GLS hydrolysis products formed, ITCs are mainly involved in direct plant defense responses (Wittstock and Burow, 2007). Indolic GLS hydrolysis differs from hydrolysis of other GLS classes since indolic ITCs are highly unstable and react with nucleophiles to form structurally diverse compounds including carbinols, whose analysis is challenging (Agerbirk et al., 2009; Chroston et al., 2022). In terms of human health, CNs and ETNs seem to be less beneficial than the corresponding ITCs (Hanschen et al., 2015; Kupke et al., 2016). CNs and ETNs are formed upon GLS hydrolysis when specifier proteins such as nitrile-specifier proteins (NSPs) or epithiospecifier proteins (ESPs) are present (Halkier and Gershenzon, 2006; Wittstock and Burow, 2010). Specifier proteins are non-heme iron proteins that convert the aglucones released by myrosinases to non-ITC products (Backenköhler et al., 2018; Eisenschmidt-Bönn et al., 2019; Mocniak et al., 2020). Their active site is located in the center of a β-propeller structure which represents the so-called Kelch domain (Gumz et al., 2015). In case of NSPs, one or two Jacalin-related lectin domains might also be present linked to the *N*-terminus of the Kelch domain (Kuchernig et al., 2012). Previous research demonstrated that NSPs, pH values less than 5 and Fe^2+^ promote CN formation at the expense of ITCs (Kissen and Bones, 2009; Wittstock and Burow, 2010). ESPs promote ETN formation from GLS-aglucones with a terminal double bond in their side chain and simple CN formation from saturated GLS-aglucones (Wittstock et al., 2016). In *A. thaliana*, functional alleles of *EPITHIOSPECIFIER MODIFIER 1* (*ESM1*) enhance ITC formation (Wittstock et al., 2016; Zhang et al., 2006). The involvement of CNs in both direct and indirect plant defense was reported, however, the biological functions of CNs and ETNs have yet to be fully elucidated (Eisenschmidt-Bönn et al., 2019; Wittstock et al., 2016). To promote ITC formation in *B. oleracea* vegetables and potentially improve plant resistance against herbivory, understanding GLS breakdown pathways involving specifier proteins is crucial (Agrawal and Kurashige, 2003; Wittstock and Burow, 2010).

*A. thaliana* possesses five NSPs (AtNSP1–AtNSP5) which are differentially expressed in the plant and have been studied biochemically with respect to their dependency on Fe^2+^ and pH as well as their activity upon hydrolysis of different GLSs. Moreover, a AtNSP1 crystal structure has been obtained (Burow et al., 2009; Kong et al., 2012; Zhang et al., 2017). In contrast, much less is known about specifier proteins from agricultural crops and vegetables of the *Brassicaceae* family. Molecular models for an NSP and an ESP encoded in the genome of broccoli (*B. oleracea* var. *italica*) have been established and used to predict possible roles of pH in stabilizing NSP–GLS aglucone interactions (Román et al., 2020). Further, we recently reported differential expression of five putative kohlrabi (*B. oleracea* var. *gongylodes*) NSPs in nine parts of mature kohlrabi using a proteomics approach (Mbudu et al., 2025). In homogenates of the nine kohlrabi parts, CN formation was predominant in the leaf midvein, leaf lamina, leaf margin, bulb core and bulb middle part. These parts had a lower ratio of myrosinase to ESP activity than the leaf stalk, bulb peel, stem and root, and low BoESM1/BoESM1-like abundance (Mbudu et al., 2025). However, so far there is no functional proof of the putative *B. oleracea* NSPs or *Brassica* NSPs in general.

Here, publicly available *B. oleracea* sequence information was used to identify sixteen putative *B. oleracea* NSPs. The evolutionary relationships among thirteen putative BoNSPs, for which complete sequences are available, were investigated, and the likely ancestral protein of the BoNSP family was identified. Multiple sequence alignment of the putative BoNSP and *Thlaspi arvense* thiocyanate forming protein (TaTFP, GenBank: AEL16674.1) sequences (Gumz et al., 2015; Kuchernig et al., 2011) revealed high conservation of the iron-binding residues (Backenköhler et al., 2018; Gumz et al., 2015) in the BoNSP candidates. Two of these NSPs, which showed contrasting abundance patterns in kohlrabi parts (Mbudu et al., 2025), were functionally characterized to prove function of NSPs in *Brassica*: the highly expressed BoNSP2 (XP_013609641.1) and root specific BoNSP11 (XP_013587057.1). The effect of Fe^2+^ concentration and the influence of pH on the activity of the recombinant BoNSP2 and BoNSP11 were investigated. Further, their activity upon hydrolysis of different GLSs was determined.

This study provides insight into the evolutionary history of the BoNSP family and offers the first evidence of functional *Brassica oleracea* NSPs. Understanding NSP function contributes towards a better understanding of the GLS hydrolysis pathway and can inform strategies used in future studies to optimize ITC formation in *Brassica* vegetables.

## 2 Materials and methods

### 2.1 Chemicals and enzymes

Allyl ITC (Allyl-ITC; ≥99%), benzonitrile (phenyl-CN, ≥99.9%), benzyl cyanide (98%), benzyl isothiocyanate (98%), 3-butenenitrile (allyl-CN, ≥98%), Coomassie brilliant blue R staining solution, D/L-dithiothreitol (DTT), iron (II) sulphate heptahydrate (FeSO_4_(H_2_O)_7_, ≥99%), isopropyl-β-d-thiogalactopyranoside (IPTG, ≥ 99%), kanamycin sulfate, myrosinase (thioglucosidase from *Sinapis alba* seeds, ≥100 units/g) and sodium carbonate (Na_2_CO_3_, ≥ 99%) were obtained from Sigma-Aldrich Chemie GmbH (Steinheim, Germany); acetic acid (supra quality, 100%), allyl GLS (sinigrin monohydrate, ≥99%), ampicillin sodium salt (≥99%), benzyl GLS (>99%), vitamin C (l-(+)-ascorbic acid, ≥99%), methylene chloride (GC Ultra Grade), NaOH (≥98%), *N*-2-hydroxyethylpiperazine-*N’*-2-ethane sulphonic acid (HEPES, ≥ 99.5%), 2-(*N*-morpholino)-ethane sulphonic acid (MES, ≥ 99%), 3-(*N*-morpholino)-propane sulphonic acid (MOPS, ≥ 99.5%), *N,N*-bis-(2-hydroxyethyl)-glycine (BICINE, ≥ 99%), sodium hydrogen carbonate (NaHCO_3_, ≥ 99.5%) were purchased from Carl Roth GmbH + Co. KG (Karlsruhe, Germany); Na_2_SO_4_ anhydrous (≥99%) was obtained from VWR International GmbH (Darmstadt, Germany); acetonitrile (LC-MS grade) was purchased from Th. Geyer GmbH & Co. KG (Renningen, Germany). 4-(Methylthio)butyl GLS (4MTB, ≥ 90%), 4-(methylsulfinyl)butyl GLS (4MSOB, ≥ 95.0%) and indol-3-ylmethyl GLS (I3M, ≥ 98%) were obtained from Phytolab GmbH and Co. KG, Vestenbergsgreuth, Germany. 4-(Methylthio)butyl ITC (4MTB-ITC, ≥ 98%) was purchased from Santa Cruz Biotechnology (Heidelberg, Germany). 5-(Methylthio)pentanenitrile (4MTB-CN) and 5-(methylsulfinyl)pentanenitrile (4MSOB-CN) (all ≥ 95% purity) were purchased from Enamine (SIA Enamine, Riga, Latvia). 4-(Methylsulfinyl)butyl ITC (4MSOB-ITC) was from Toronto Research Chemicals (Toronto, Canada). Gateway™ LR clonase™ II enzyme mix, GeneJET gel extraction kit, pENTR-D/TOPO, Pierce™ Bradford protein assay kit and indole-3-carbonitrile (I3N, 98%) were obtained from Thermo Fisher Scientific (Germany). The Gateway compatible pMAL-c2 was from New England Biolabs.

MS grade solvents and ultrapure water were utilized in all experiments.

### 2.2 Database search and domain prediction

The *A. thaliana NSP1* (*At3g16400.1*), *NSP2* (*At2g33070.3*), *NSP3* (*At3g16390.1*), *NSP4* (*At3g16410.1*) and *NSP5* (*At5g48180.1*) nucleotide and amino acid sequences were retrieved from TAIR (https://www.arabidopsis.org/) and used as a query against the *B. oleracea* var. *oleracea* genome at NCBI using the BLASTn and BLASTp search tools (https://blast.ncbi.nlm.nih.gov/Blast.cgi). The BLASTp searches were done against the non-redundant protein sequences database (nr). The protein sequences of the putative BoNSPs identified at NCBI were used as a query against the *B. oleracea* genome at Ensembl Plants with the BLASTX search tool (https://plants.ensembl.org/Brassica_oleracea/Tools/Blast). The Kelch domains in the putative BoNSPs were predicted manually based on multiple sequence alignment with the full amino acid sequence of TaTFP (Gumz et al., 2015; Kuchernig et al., 2011). The amino acid sequences upstream of the predicted Kelch domain regions were extracted and the jacalin domains were predicted by InterPro 106.0 (Blum et al., 2024) (released on 19 June 2025). The Kelch and jacalin domains in the putative BoNSPs were schematically depicted using IBS2.0 (Xie et al., 2022). The amino acid sequence identity between the putative BoNSP and AtNSP isoforms was assessed by multiple sequence alignment of their predicted Kelch domain sequences using ClustalW in Clustal Omega (Madeira et al., 2024) and visualized in Jalview version 2.11.4.1 (Waterhouse et al., 2009). The molecular weight of XP_013583566.1 and XP_013589780.1 not detected in Ensembl Plants was calculated using ExPASy ProtParam (Gasteiger et al., 2005).

### 2.3 Phylogenetic analysis of the candidate BoNSPs

To investigate the evolutionary history of the Kelch domain region, the amino acid sequences of the putative BoNSPs and AtNSPs were trimmed manually to obtain the sequence parts representing only the Kelch domain region. The Kelch domain regions of these specifier proteins as well as the putative ancestral proteins (At3g07720 and *B. oleracea* homolog) were aligned using the L-INS-i iterative refinement method in MAFFT version 7 (Katoh et al., 2019; Kuraku et al., 2013) and the phylogenetic tree was constructed using the Maximum likelihood algorithm and JTT matrix-based model with 1000 bootstrap replicates in MEGA11 (Tamura et al., 2021) using *Vitis vinifera* XP_002267128.1 as an outgroup. The Maximum Parsimony method was automatically applied to generate the initial tree(s) for the heuristic search. The differences in evolutionary rates among different sites were modelled using a discrete Gamma distribution (5 categories (+G, parameter = 1.3173)). Twenty sequences were analyzed and the complete deletion option was applied to remove all positions with gaps and missing data leaving a final dataset with 304 positions.

**Figure 1:**
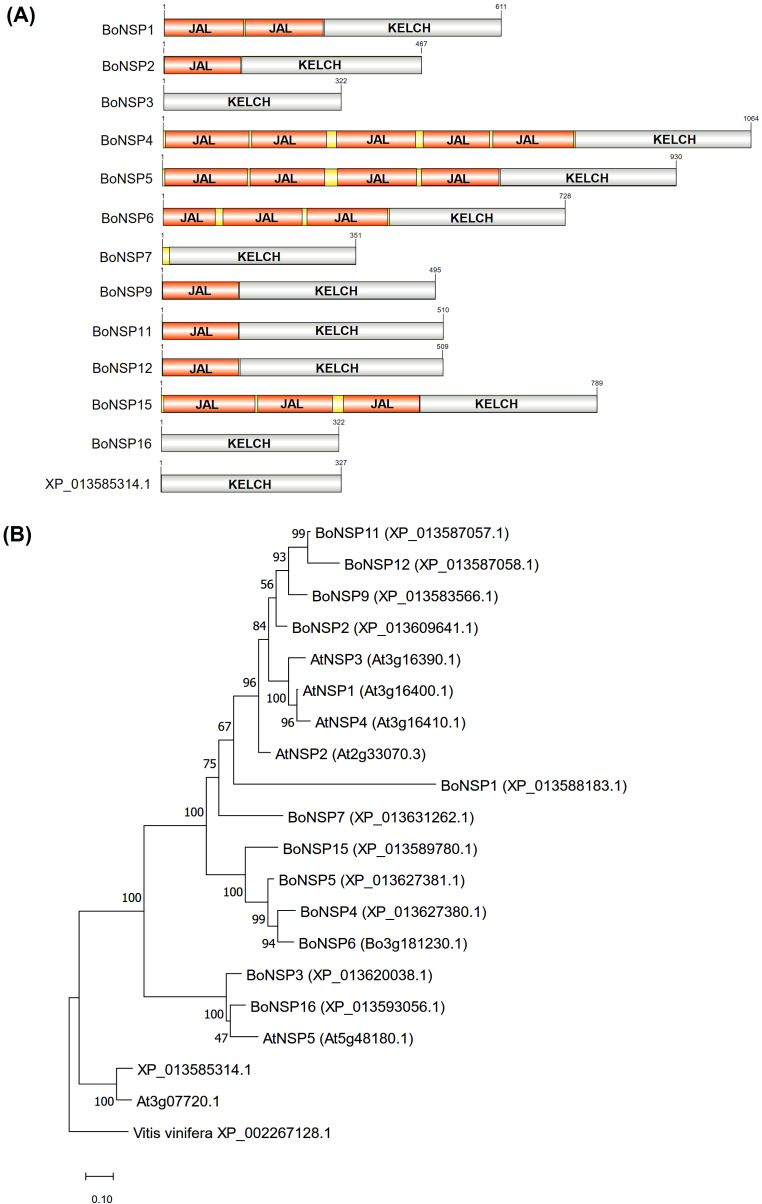
Domain structure and phylogenetic relationship of BoNSPs. **(A)** Schematic depiction of the Kelch domains (KELCH) predicted based on multiple sequence alignment with TaTFP (Gumz et al., 2015; Kuchernig et al., 2011) and the *N-*terminal jacalin-like lectin domains (JAL, IPR001229) predicted by InterPro (version 106.0) (Blum et al., 2024). The domains in the candidate BoNSPs were visualized by IBS 2.0 (Xie et al., 2022). **(B)** Phylogenetic analysis of the putative *B*. *oleracea* NSPs based on their amino acid sequences. The evolutionary history was inferred using the Maximum Likelihood method based on the JTT matrix-based model (Jones et al., 1992) with 1000 bootstrap replicates in MEGA11 (Tamura et al., 2021). The tree with the highest log likelihood (-5496.92) is shown. The bootstrap values are shown next to the branches. The tree is drawn to scale and the branch lengths correspond to the number of substitutions per site.

The jacalin domains were used separately for phylogenetic analysis. The jacalin domains, predicted by InterPro (Blum et al., 2024), were manually extracted, supported by the Group Protein tool in Sequence Manipulation Suite (Stothard, 2000) to locate the jacalin regions. For BoNSP candidates with more than one jacalin domain, the domains were assigned single-letter codes as done previously (Burow et al., 2009; Kuchernig et al., 2012), but starting from the *C*-terminus and analyzed separately. A total of twenty-one jacalin domains from the putative BoNSPs were identified. A significantly shorter jacalin domain (BoNSP6c_(Bo3g181230.1), 94 amino acids) (**Figure 1A** and **Supplementary Table S4**) was excluded from the phylogenetic analysis. To select representative jacalin domains for further analysis, the jacalin domains were first aligned as was done for the Kelch domains. The alignment was then used to construct a phylogenetic tree using the Neighbor-joining algorithm with 1000 bootstrap replicates in MEGA11 (Tamura et al., 2021). To search for related myrosinase binding proteins encoded in the *B. oleracea* genome, six jacalin domains ((BoNSP1b_(XP_013588183.1), BoNSP4b_(XP_013627380.1), BoNSP5b_(XP_013627381.1), BoNSP5a_(XP_013627381.1), BoNSP12_(XP_013587058.1) and BoNSP15b_(XP_013589780.1)) (**Supplementary Table S4**) were selected as representatives of the twenty jacalin domains based on their separate grouping in an initial Neighbour-joining tree. BLASTp searches using the six jacalin domain sequences identified three putative BoMBP2 isoforms (designated as BoMBP2-1, XP_013609657.1; BoMBP2-2, XP_013609679.1 and BoMBP2-3, XP_013624973.1) in the *B. oleracea* var. *oleracea* genome at NCBI (**Supplementary Table S4**). Using the three putative BoMBP2 sequences, three BoMBP orthologues were identified in the *Oryza sativa Japonica* Group (rice) genome and two in the *Populus trichocarpa* (poplar) genome. In all the BLAST searches, the nr database was searched and only the protein that appeared as the top hit was retrieved, except for rice, where a hypothetical protein (GenBank: EEE64302.1), which appeared as a top hit for the putative BoMBP2-3 (XP_013624973.1), was excluded, and the jacalin-related lectin isoform X1 protein (XP_066166746.1), which emerged as the second hit, was retrieved. The jacalin domain sequences from the candidate BoNSPs and AtNSPs (analyzed separately if the protein had more than one jacalin domain), the full-length protein sequences of the putative BoMBP isoforms, the BoMBP orthologues from rice and poplar, AtMBP1 (At1g52040.1), AtMBP2 (At1g52030.1), and *Oryza sativa Japonica* Group NP_001396161.1, used as an outgroup, were aligned using the iterative refinement method L-INS-i in MAFFT (Katoh et al., 2019; Kuraku et al., 2013). The phylogenetic tree was constructed using the Maximum likelihood algorithm and WAG + G (Whelan and Goldman, 2001) model with 1000 bootstraps in MEGA11 (Tamura et al., 2021). The Maximum Parsimony method was automatically applied to generate the initial tree(s) for the heuristic search. Moreover, the differences in evolutionary rates among different sites were modelled using a discrete Gamma distribution (5 categories (+G, parameter = 2.7148)). Thirty-six sequences were analyzed and the partial deletion option was applied to remove all positions with less than 95% site coverage, leaving a final dataset with 127 positions.

### 2.4 Plasmid construction, heterologous expression and purification of BoNSP2 and BoNSP11

The BoNSP2 (XP_013609641.1) and BoNSP11 (XP_013587057.1) cDNAs were commercially synthesized (Eurofins Genomics GmbH, Cologne, Germany). The cDNAs were amplified using gene-specific primers (listed in **Supplementary Table S1**), cloned into the pENTR-D/TOPO Gateway entry vector and confirmed by whole plasmid sequencing (Eurofins Genomics GmbH, Cologne, Germany). For protein expression, the coding sequences were then recombined into the Gateway-compatible version of pMAL-c2 with an *N*-terminal maltose-binding protein-tag. The recombinant BoNSP2 and BoNSP11 were expressed in *Escherichia coli* BL21 cells by overnight induction in LB medium with 0.5 mM isopropyl β-d-1-thiogalactopyranoside at 220 rpm and 16 °C. The bacterial cultures were then centrifuged for 15 min at 4000 g and 4 °C, the supernatant discarded, the bacteria pellet lysed by sonication and the heterologously expressed proteins purified using amylose resin following the manufacturer’s instructions. Recombinant proteins were eluted with maltose binding protein elution buffer composed of 20 mM Tris-HCl (pH 8), 150 mM NaCl, 1 mM dithiothreitol (DTT) and 20 mM maltose. Protein expression and purity of the eluted proteins were assessed using SDS-PAGE followed by Coomassie blue staining. The Bradford protein assay (Bradford, 1976), using bovine serum albumin as a standard, was used to determine the concentration of the eluted proteins. After adjustment of the protein concentration to 0.5 µg/µl in deionized water and glycerol added to a final concentration of 10% (v/v), the proteins were flash-frozen in liquid nitrogen and stored at – 80 °C.

### 2.5 NSP assay and GC-MS analysis of GLS hydrolysis products

The protocol used previously (Witzel et al., 2019) to determine the BoESP activity was slightly adapted for NSP activity. NSP activity was assessed in assays containing 50 µl of purified recombinant BoNSP2 or BoNSP11 (25 µg) mixed with 10 µl of 25 mM vitamin C solution, 50 µl of 10 mM allyl GLS solution and 350 µl of 50 mM sodium acetate (NaAc) buffer (pH 5.5) containing 1 mM DTT and 50 µM Fe^2+^ (added as iron (II) sulphate heptahydrate). All reactions with BoNSP2 were started by addition of 50 µl of 0.5 U/ml *Sinapis alba* myrosinase. Similarly, all reactions with BoNSP11 were started by the addition of 50 µl of 0.28 U/ml *Sinapis alba* myrosinase. According to the manufacturer, 1 U was defined as the quantity of *Sinapis alba* myrosinase which catalyzes the production of 1 µmol of glucose per minute from allyl GLS at pH 6 and 25 °C. The units of myrosinase used for the assays with BoNSP2 and BoNSP11 were calculated based on the enzymatic activity specified for each batch by the manufacturer (Batch BCBQ2804V – 495 U/g for BoNSP2 and Batch BCCG4678 – 279.6 U/g for BoNSP11). After 1 h of incubation at 25 °C, internal standard (0.2 µmol phenyl-CN) was added and the GLS hydrolysis products were extracted thrice using methylene chloride and analyzed by a gas chromatography - mass spectrometry (GC-MS) system (7890 A GC with 5975C Inert XL MSD, Agilent Technologies Deutschland GmbH, Waldbronn, Germany) using an HP-5MS Ultra Inert column (30 m x 0.25 mm x 0.25 μm; Agilent Technologies, Waldbronn, Germany) as described previously (Hanschen, 2024). Since CNs are also formed in the absence of NSPs, controls containing all reaction components except BoNSPs were conducted. NSP activity was assessed as the % allyl-CN relative to all detected allyl GLS hydrolysis products (Allyl-CN, Allyl-ITC) and calculated as follows: (% Allyl-CN = ([Allyl-CN]/([Allyl-ITC] + [Allyl-CN]) * 100%). This protocol was modified for the different analyses carried out in this study, and the specific details are outlined in the following sections. Each BoNSP was expressed, purified and characterized three times independently.

### 2.6 Effect of ferrous ion concentration on BoNSP2 and BoNSP11 activity

To assess the effect of ferrous ion (Fe^2+^ - added as iron (II) sulphate heptahydrate) concentration on BoNSP activity, the NSP assay was performed as described above with some modifications. For BoNSP2, the assays were performed without and with Fe^2+^ supplementation to a final concentration of 6.86 µM, 13.7 µM, 34.3 µM, 68.6 µM, 137 µM and 343 µM. To determine whether BoNSP2 activity is strictly dependent on the added iron, EDTA was added to a final concentration of 250 µM and 500 µM in assays containing 34.3 µM Fe^2+^. The experiment was also conducted for BoNSP11 but using only 34.3 µM Fe^2+^ and 343 µM Fe^2+^. The effect of EDTA was tested at 250 µM in assays containing 34.3 µM Fe^2+^.

### 2.7 Activity of BoNSP2 and BoNSP11 using selected GLSs

NSP activity was investigated as described above using allyl GLS, 4-(methylthio)butyl GLS (4MTB GLS), 4-(methylsulfinyl)butyl GLS (4MSOB GLS), benzyl GLS and indol-3-ylmethyl GLS (I3M GLS) as substrates for myrosinase. The fold-increase in CN formation was calculated by dividing the amount of CNs (in µmols) recovered in the assay with BoNSP divided by the CNs (in µmols) recovered in the control assays with all reaction components except the recombinant BoNSP.

### 2.8 Influence of pH on BoNSP activity

To investigate the influence on pH on BoNSP2 activity, the NSP assay was performed as described above but with six buffers, NaAc, 2-(*N*-morpholino)-ethane sulphonic acid (MES), 3-(*N*-morpholino)-propane sulphonic acid (MOPS), *N*-2-hydroxyethylpiperazine-*N’*-2-ethane sulphonic acid (HEPES), *N,N*-bis-(2-hydroxyethyl)-glycine (BICINE) and carbonate-bicarbonate buffer (Na_2_CO_3_/NaHCO_3_), with overlapping pH ranges. The final concentration of all six buffers in each sample was 34.3 mM and the pH values used for each buffer were as follows: NaAc buffer pH 5.5; MES buffer pH 5.5, pH 6 and pH 6.5; MOPS buffer pH 6.5, pH 7 and pH 7.5; HEPES buffer pH 7.5 and pH 8; BICINE buffer pH 8, pH 8.5 and pH 9, and Na_2_CO_3_/NaHCO_3_ buffer pH 9, pH 9.5, pH 10 and pH 10.5.

Although NaAc has an effective pH range of 3.7 to 5.6, the sensitivity of BoNSP2 and BoNSP11 to changes in pH was also assessed in NSP assays set up as outlined above but with 50 µl of a 12 mM allyl GLS solution and NaAc solution set from pH 4 to pH 12 (for BoNSP2) and pH 4 to pH 9 (for BoNSP11), by the addition of acetic acid or NaOH.

### 2.9 Statistical analysis

Statistical analysis was performed using SigmaPlot for Windows Version 14.0, Build 14.0.3.192 (Systat Software, Inc., San Jose, CA, USA). Normal distribution was assessed using the Shapiro-Wilk test and the homogeneity of variance by the Brown-Forsythe test. If normality and homogeneity of variance were confirmed, one-way ANOVA followed by Tukey’s *posthoc* test (*p≤0.05*) was used. If normality or homogeneity of variance, or both, were not confirmed, the non-parametric Kruskal−Wallis ANOVA followed by Dunn’s *post-hoc* test was used instead. Homogeneous groups were assigned using the *multcompLetters()* function in R (v4.4.3) (Posit Software, PBC, Boston, MA, USA).

## 3 Results

### 3.1 Identification of putative *B. oleracea* NSPs

In a first approach, a search for putative BoNSPs was performed in the *B. oleracea* var. *oleracea* genome at NCBI using nucleotide and amino acid sequences of AtNSP1–AtNSP5 as a query. The retrieved amino acid sequences of putative BoNSPs were then used for a search in the Ensembl Plants database. From the database search, a total of seventeen BoNSP candidates were identified. The number was revised to sixteen (**Table 1**) after identification and exclusion of the likely ancestral protein of the BoNSP family through phylogenetic analysis, which will be detailed in the following section. Five of the sixteen BoNSP isoforms were previously found to be expressed in mature kohlrabi (Mbudu et al., 2025) (**Table 1**). Two proteins encoded in the *B. oleracea* var. *oleracea* genome and previously annotated as myrosinase-binding protein 2-like (isoforms XP_013627380.1 and XP_013627381.1) were also designated as putative BoNSPs as they possess *N*-terminal jacalin-like lectin domains and Kelch domains, which is characteristic of NSPs (Burow et al., 2009). Two candidates (XP_013583566.1 and XP_013589780.1) were only found at NCBI (**Table 1**). From Ensembl Plants, two additional proteins (Bo3g181230.1 and Bo5g126040.1) with significant sequence similarity to the BoNSP candidates from NCBI were identified. Protein Bo5g125020.1 in Ensembl Plants, which has a length of 542 amino acids, starts with a proline suggesting an inaccurate gene model. Therefore, Bo5g125020.1 was not included in further analyses. However, protein XP_013583566.1 in NCBI was 100% identical to the 495 *C*-terminal amino acids of Bo5g125020.1, indicating that this sequence is derived from the same genomic position (**Table 1**). Similarly, the genomic position of XP_013589780.1 was determined based on the genomic position of proteins with partial sequence identity found in Ensembl Plants: (1) Bo6g027570.1, 100% identical to the *C*-terminal 287 amino acids of XP_013589780.1, and (2) Bo6g027580.1, 100% identical to the *N*-terminal 478 amino acids of XP_013589780.1 (**Table 1**). Thirteen putative BoNSPs (XP_013588183.1, XP_013609641.1, XP_013620038.1, XP_013627380.1, XP_013627381.1, Bo3g181230.1, XP_013631262.1, XP_013583566.1, XP_013587057.1, XP_013587058.1, XP_013585314.1, XP_013589780.1 and XP_013593056.1) for which complete protein sequences were obtained based on seemingly correct gene models and whose genomic position could be assigned with sufficient certainty (**Table 1**) were analyzed in more detail.

**Table 1:** Annotation of the sixteen candidate *B. oleracea* NSPs identified in the database search at NCBI or Ensembl Plants.

Protein	NCBI gene ID	NCBI protein ID		Ensembl plants ID	CDS (aa)	Notes
BoNSP1	LOC106296567		XP_013588183.1	Bo1g123040.1	611	
BoNSP2	LOC106316308		XP_013609641.1	Bo1g123520.1	467	Designated as BoNSP2 in our previous study (Mbudu et al., 2025) Has 100% amino acid sequence identity to the broccoli NSP modelled *in silico* (Román et al., 2020)
BoNSP3	LOC106326669		XP_013620038.1	Bo2g149730.1	322	
BoNSP4	LOC106333487		XP_013627380.1	Bo3g181200.1	1064	Formerly assigned as myrosinase-binding protein 2-like
BoNSP5	LOC106333488		XP_013627381.1	Bo3g181210.1	930	Formerly assigned as myrosinase-binding protein 2-like
BoNSP6	–		–	Bo3g181230.1	728	
BoNSP7	LOC106336851		XP_013631262.1	Bo4g178080.1	351	Designated as BoNSP2 in our previous study (Mbudu et al., 2025)
BoNSP8	–		–	Bo5g125020.1	542	Gene model presumably incorrect
BoNSP9	LOC106292512		XP_013583566.1	–	495	Identical to 495 *C*-terminal amino acids of Bo5g125020.1
BoNSP10	–		–	Bo5g126040.1	259	Presumably incomplete protein sequence
BoNSP11	LOC106295641		XP_013587057.1	Bo5g126100.1	510	Designated as BoNSP1 in our previous study (Mbudu et al., 2025)
BoNSP12	LOC106295642		XP_013587058.1	Bo5g126120.1	509	
n. a.	LOC106294305		XP_013585314.1	Bo5g141230.1	327	Designated as BoNSP5 in our previous study (Mbudu et al., 2025)/ancestral protein (NSP progenitor) based on phylogenetic analysis.
BoNSP13	–		–	Bo6g027570.1	287	Presumably incomplete protein sequence/identical to 287 *C*-terminal amino acids of XP_013589780.1
BoNSP14	–		–	Bo6g027580.1	482	Presumably incomplete protein sequence/identical to 478 *N*-terminal amino acids of XP_013589780.1
BoNSP15	LOC106298243		XP_013589780.1	–	789	
BoNSP16	LOC106301248		XP_013593056.1	Bo7g089110.1	322	Designated as BoNSP5 in our previous study (Mbudu et al., 2025)

The five putative NSP isoforms identified in parts of mature kohlrabi in our previous study (Mbudu et al., 2025) are indicated (see Notes column). The dash (*–*) indicates protein isoforms not detected in the respective database. CDS (aa) refers to the number of amino acids (aa) deduced from the coding sequence (CDS). n. a., not applicable.

Based on the information in Ensembl Plants and NCBI, the thirteen putative BoNSPs selected for further analyses, consist of 322 to 1064 amino acids (**Table 1**) and have a molecular weight of 34.8 kDa to 116.4 kDa. Manual prediction of the Kelch domains based on the sequence of TaTFP (GenBank: AEL16674.1) (Gumz et al., 2015; Kuchernig et al., 2011) (**Supplementary Figure S2**) and prediction of the jacalin domains by InterPro (Blum et al., 2024) revealed that all candidate BoNSPs were composed of a Kelch domain and nine of them possessed up to five jacalin-like lectin domains at the *N*-terminus of their Kelch domain (**Figure 1A**).

### 3.2 Phylogenetic relationship of BoNSPs

To elucidate the evolutionary relationships among the putative BoNSPs, a phylogenetic tree based on the Maximum likelihood algorithm was generated from the Kelch domains of the thirteen putative BoNSPs, five AtNSPs (AtNSP1–AtNSP5), At3g07720.1, the ancestor of the AtNSPs (Burow et al., 2009), and *Vitis vinifera* XP_002267128.1 as an outgroup (**Figure 1B**). At the base of the tree, XP_013585314.1 grouped together with At3g07720.1 (**Figure 1B**). As At3g07720.1 does not seem to possess specifier protein activity (Burow et al., 2009), this suggests that XP_013585314.1 represents the corresponding ancestral protein in *B. oleracea* and may have its primary role outside of the GLS-myrosinase system. XP_013585314.1 was therefore not designated as a putative BoNSP. Both At3g07720.1 and XP_013585314.1 have only the Kelch domain, supporting the hypothesis that the presence of jacalin domains in NSPs may be a derived state (Burow et al., 2009; Kuchernig et al., 2012). Having excluded XP_013585314.1 as a candidate BoNSP, the rest of the putative BoNSPs were assigned consecutive numbers (BoNSP1–BoNSP16) (**Table 1**) based on their genomic position inferred from their Ensembl Plants IDs (**Table 1**). This resulted in a different numbering scheme than previously used (Mbudu et al., 2025). The new and the previous names are indicated in **Table 1**. Multiple sequence alignment of the Kelch domains from the thirteen putative BoNSPs included in the phylogenetic analysis (BoNSP1–BoNSP7, BoNSP9, BoNSP11, BoNSP12, BoNSP15 and BoNSP16) (**Table 1**) and AtNSPs using Clustal Omega, revealed 40.1% to 89.4% amino acid sequence identity among putative BoNSPs and 40.2% to 86.5% sequence identity with the AtNSPs (**Supplementary Table S3**).

The other branch of the phylogenetic tree had two major clades: one with AtNSP5 as well as BoNSP3 and BoNSP16 and another one with two subclades. All proteins in the AtNSP5 clade lack a jacalin domain. In spite of the low branch support for AtNSP5, the grouping of BoNSP3 and BoNSP16 with AtNSP5 might indicate that proteins in this clade are the oldest functional NSPs (Kuchernig et al., 2012). In contrast, all proteins in the other major clade (AtNSPs and putative BoNSPs, except BoNSP7) possess one or several jacalin domains (**Figure 1A**). Although the phylogenetic tree was built using only the Kelch domain regions, the BoNSP candidates in the two subclades of this major clade group according to their numbers of jacalin domains. One subclade contains the four BoNSP candidates with three to five jacalin domains (BoNSP4–BoNSP6 and BoNSP15) and no AtNSPs. The other subclade comprises six BoNSP candidates (BoNSP1, BoNSP2, BoNSP7, BoNSP9, BoNSP11 and BoNSP12) with a maximum of two jacalin domains (**Figure 1A**) and AtNSP1–AtNSP4 with one or two jacalin domains. Although the two subclades have a high bootstrap support, the positioning of BoNSP7 in the tree is surprising as this is the only BoNSP in the clade that lacks a jacalin domain.

Past reports suggest that the jacalin domains in AtNSPs may derive from MBPs and may be from different sources (Burow et al., 2009; Kuchernig et al., 2012). To test whether this is likely for the putative BoNSPs, phylogenetic analysis of the jacalin domains was performed similarly to what has been described before (Burow et al., 2009; Kuchernig et al., 2012). A total of twenty-one jacalin domains from nine candidate BoNSPs (BoNSP1, BoNSP2, BoNSP4–BoNSP6, BoNSP9, BoNSP11, BoNSP12, and BoNSP15) (**Figure 1A** and **Supplementary Table S4**) were identified. If a BoNSP candidate had more than one jacalin domain, domains were assigned single-letter codes (a–e), starting at the *C*-terminus of the protein. One jacalin domain (BoNSP6c_(Bo3g181230.1), 94 amino acids), which was significantly shorter than the average length of the twenty-one jacalin domains (139 amino acids) (**Figure 1A** and **Supplementary Table S4**), was excluded from the phylogenetic analysis. A phylogenetic tree was generated from the twenty putative BoNSP and five AtNSP jacalin domain sequences, and the full-length protein sequences of the three putative BoMBP2 isoforms (designated as BoMBP2-1, XP_013609657.1; BoMBP2-2, XP_013609679.1 and BoMBP2-3, XP_013624973.1), three BoMBP2 orthologues in rice and two in poplar (**Supplementary Table S4**), AtMBP1 (At1g52040.1) and AtMBP2 (At1g52030.1) (**Supplementary Figure S5**). The tree topology strongly supported the branching off of the *B. oleracea* and *A. thaliana* sequences from the rice and poplar sequences. The *B. oleracea* and *A. thaliana* sequences formed two well supported clusters. One cluster (cluster A) was composed of sequences from both species and contained the jacalin domains located directly upstream of the Kelch domain region of all included putative BoNSPs and AtNSPs. In addition, several further jacalin domains (second, third or fourth jacalin domains upstream of the BoNSP Kelch domain region and jacalin domain “a” of AtNSP4) as well as AtMBP1, AtMBP2 and two BoMBP2 isoforms (BoMBP2-2 (XP_013609679.1) and BoMBP2-3 (XP_013624973.1)) belonged to this cluster. The other cluster (cluster B) was confined to *B. oleracea* sequences and contained only BoNSP jacalin domains located not directly adjacent to the Kelch domain region as well as BoMBP2 isoform BoMBP2-1 (XP_013609657.1). Again, this included second, third, fourth or fifth jacalin domains upstream of the BoNSP Kelch domain region. Thus, the jacalin domains directly adjacent to the Kelch domain of all included NSP sequences appear to share a common origin and are closely related to AtMBP1, AtMBP2, and BoMBP2 isoforms BoMBP2-2 (XP_013609679.1) and BoMBP2-3 (XP_013624973.1). The additional jacalin domains are either closely related to the first ones (same cluster, A) or the result of an early gene duplication in *B. oleracea* (or the genus *Brassica*) leading to cluster B with BoMBP2 isoform BoMBP2-1 (XP_013609657.1). Jacalin domains of NSPs with only one or two jacalin domains of both *B. oleracea* and *A. thaliana* were present only in cluster A while jacalin domains of NSPs with more than two jacalin domains were present across clusters A and B (**Supplementary Figure S5**). Bootstrap support within the clusters was too low to draw further conclusions. Adjusting the parameters used for the multiple sequence alignment in MAFFT (Katoh et al., 2019; Kuraku et al., 2013) and for constructing the phylogenetic tree in MEGA (Tamura et al., 2021) did not make the phylogenetic tree more robust.

### 3.3 BoNSP2 and BoNSP11 are nitrile-specifier proteins whose activity is affected by added ferrous ions

**Figure 2:**
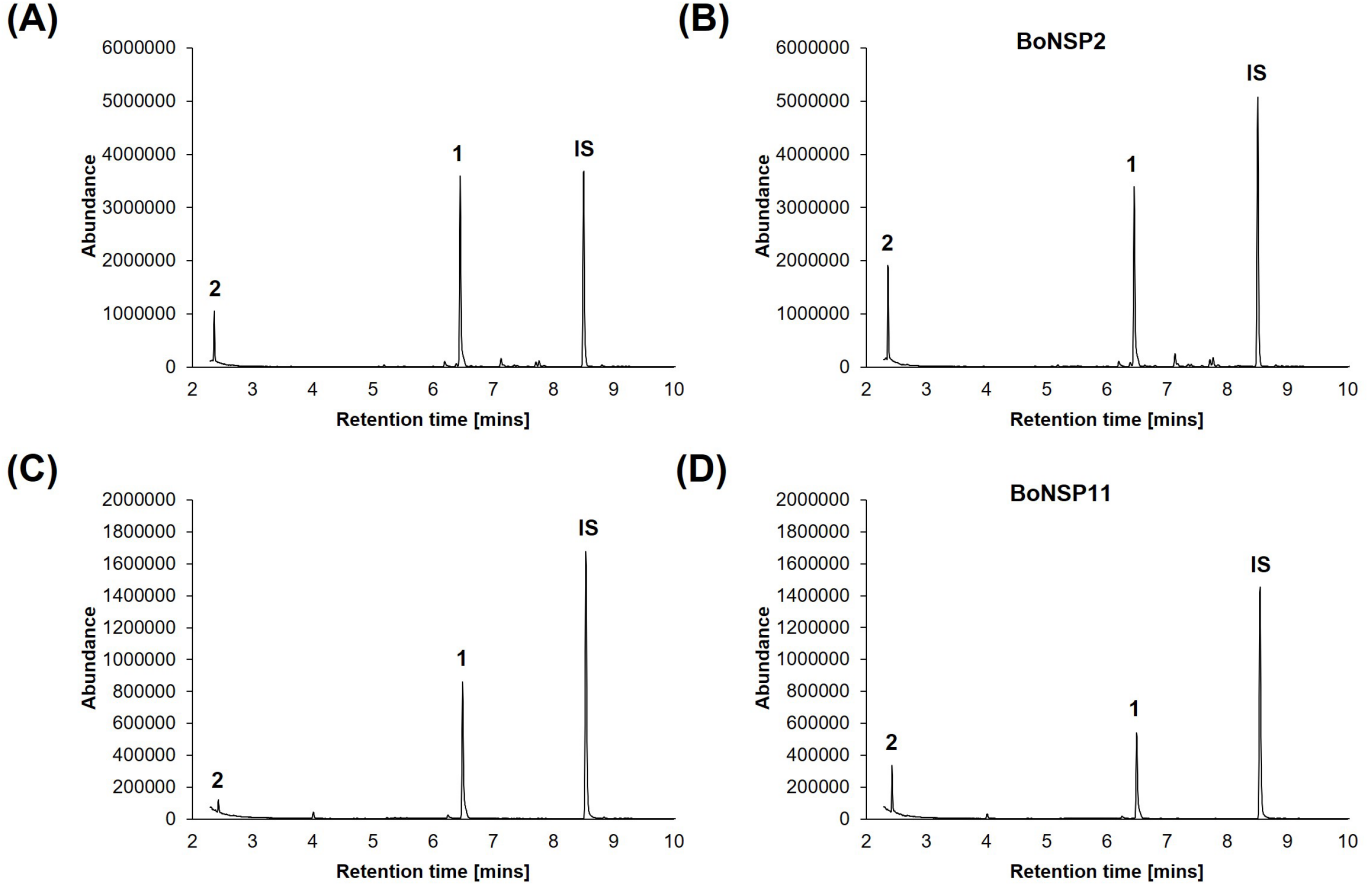
The effect of purified recombinant BoNSP2 and BoNSP11 on the formation of allyl-CN from allyl GLS *in vitro*. The gas chromatography (GC) traces (total ion current) depict the allyl GLS hydrolysis products formed in NSP assays after 1 h incubation of 0.98 mM allyl GLS with *Sinapis alba* myrosinase and **(A)** 6.86 µM Fe^2+^**(B)** purified BoNSP2 (25 μg) and 6.86 µM Fe^2+^**(C)** 34.3 µM Fe^2+^ and **(D)** purified BoNSP11 (25 μg) and 34.3 µM Fe^2+^. The peaks represent the allyl GLS hydrolysis products detected: 1 allyl-ITC, and 2 allyl-CN and IS – internal standard (phenyl-CN).

BoNSP2 (XP_013609641.1) and BoNSP11 (XP_013587057.1) with contrasting abundance patterns in kohlrabi parts (Mbudu et al., 2025) were further characterized. BoNSP2 (XP_013609641.1) was detected in all mature kohlrabi parts analyzed excluding the leaf lamina whereas BoNSP11 (XP_013587057.1) was solely detected in the root (Mbudu et al., 2025). The alignment of the peptides identified in our previous study (Mbudu et al., 2025) with the complete protein sequence revealed a sequence coverage of 48.2% (BoNSP2, 20 peptides) and of 10.4% (BoNSP11, 6 peptides) (**Supplementary Figures S3**, **S4** and **Supplementary Table S2**). To determine whether the BoNSPs are functional, their NSP activity was investigated *in vitro* using purified recombinant proteins. The non-enzymatic formation of CNs from GLS hydrolysis *in vitro* has been reported before (Burow et al., 2009; Kong et al., 2012). As expected, some allyl cyanide (allyl-CN) was formed in assays with all the reaction components except BoNSP2 (**Figure 2A**) or BoNSP11 (**Figure 2C**). The proportion of allyl-CN formed upon GLS hydrolysis increased in the presence of BoNSPs as depicted here for assays containing 6.86 µM Fe^2+^ and BoNSP2 (**Figure 2B**), and 34.3 µM Fe^2+^ and BoNSP11 (**Figure 2D**), confirming the NSP activity of BoNSP2 and BoNSP11 *in vitro*.

**Figure 3:**
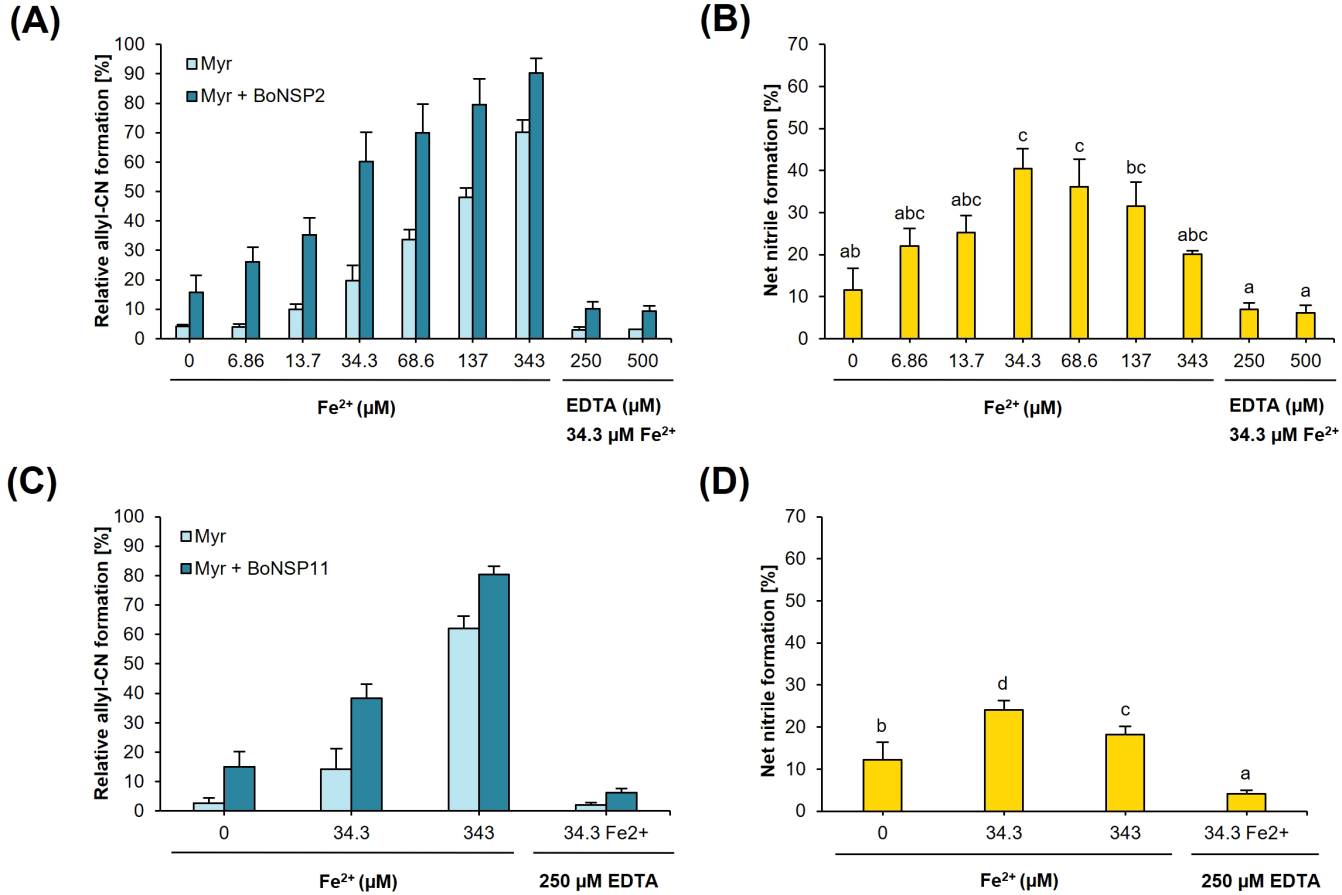
Effect of ferrous ion (Fe^2+^) concentration on the *in vitro* activity of BoNSP2 (XP_013609641.1) **(A, B)** and BoNSP11 (XP_013587057.1) **(C, D)**. The activity was assessed as the proportion of allyl-CN [%] formed relative to the total allyl GLS hydrolysis products in assays with 0.98 mM allyl GLS incubated with *Sinapis alba* myrosinase alone (Myr, control) or both myrosinase and **(A)** BoNSP2 (Myr + BoNSP2) or **(C)** BoNSP11 (Myr + BoNSP11). The net nitrile formation [%] for BoNSP2 and BoNSP11 was calculated by subtracting the proportion of allyl-CN formed in the control reactions with myrosinase alone (Myr) from the proportion of nitriles formed in assays with myrosinase and **(B)** BoNSP2 (Myr + BoNSP2) or **(D)** BoNSP11 (Myr + BoNSP11). Values shown are the means ± SD of three independent expression experiments (n = 3). The lowercase letters indicate a significant difference in net nitrile formation at different iron concentrations in the presence of **(B)** BoNSP2 as determined by Kruskal−Wallis-ANOVA and Dunn’s *post-hoc* test (H = 43.838, *p*<0.001) and **(D)** BoNSP11 as determined by one-way ANOVA followed by Tukey’s *post-hoc* test (*p≤*0.05).

Next, we tested, at which concentration of added Fe^2+^ the highest proportion of CN is formed from allyl GLS in BoNSP-myrosinase reaction mixtures. Addition of Fe^2+^ to a final concentration of up to 343 µM led to an increase of the proportion of allyl-CN formed from allyl GLS in both the control assays as well as in BoNSP2 (**Figure 3A**) or BoNSP11 (**Figure 3C**) containing assays. Net CN formation was obtained by subtracting CN proportion in controls from that in reactions containing BoNSPs. Net CN formation increased from 11.6% in assays with no Fe^2+^ supplementation to 40.5% at 34.3 µM Fe^2+^ in assays with BoNSP2 and from 12.3% to 24.1% in assays with BoNSP11 (**Figures 3B, D** and **Supplementary Table S5**). Highest net CN formation was observed in assays containing 34.3 µM Fe^2+^ (**Figures 3B, D** and **Supplementary Table S5**). When EDTA at 250 µM or 500 µM was added to the reactions containing 34.3 µM Fe^2+^, net CN formation in reactions with or without BoNSP2 and BoNSP11 was significantly reduced (**Figures 3B, D**), but not completely abolished. Thus, both the background CN formation by myrosinase and CN formation by BoNSPs did not strictly depend on added iron.

### 3.4 BoNSP2 and BoNSP11 differ in their activity upon hydrolysis of different GLSs

**Figure 4:**
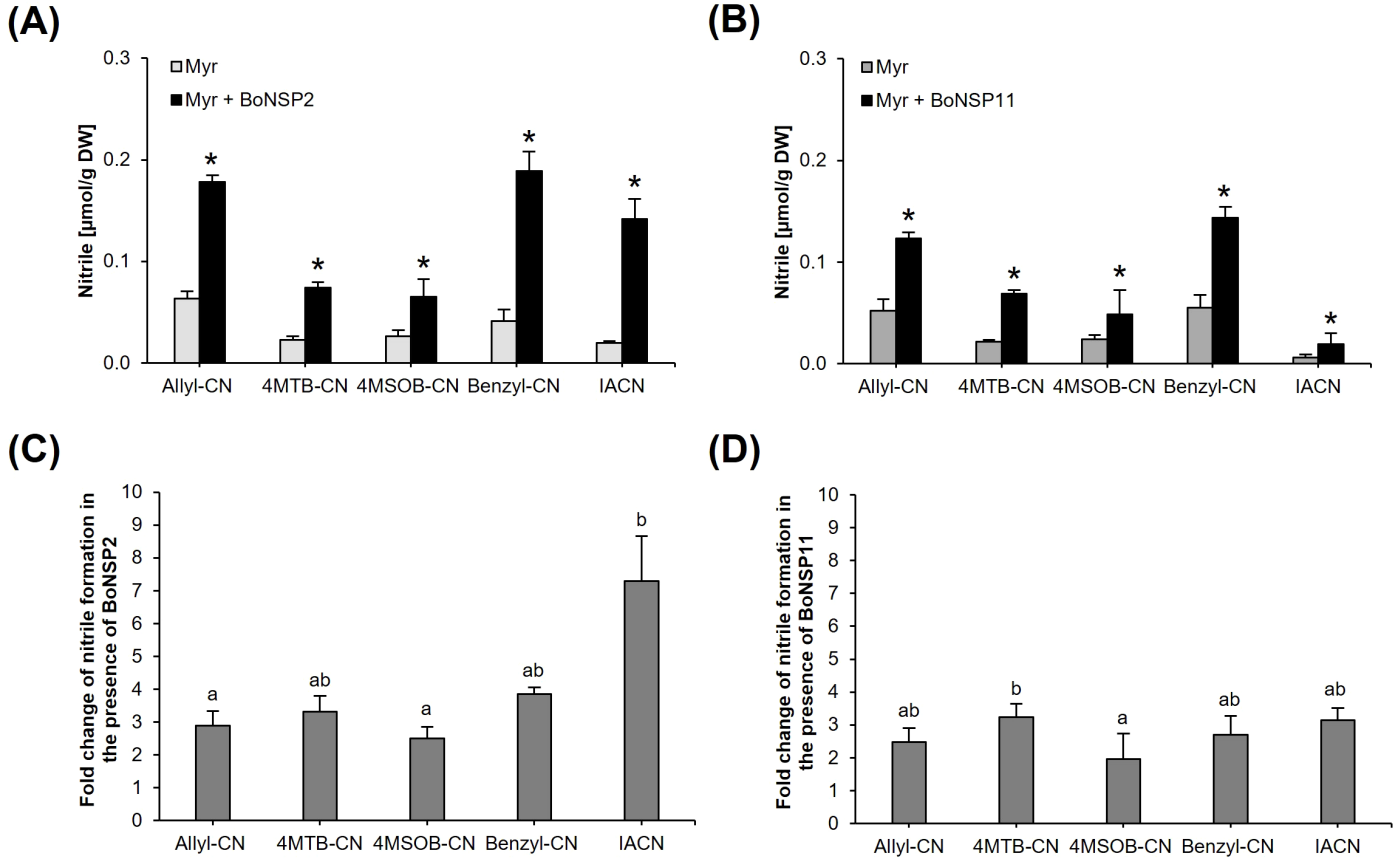
Hydrolysis activity of the recombinant BoNSP2 (XP_013609641.1) and BoNSP11 (XP_013587057.1) isoforms assessed as the increase in nitrile formation (in µmol/g DW) from five pure GLS standards (allyl GLS, 4MTB GLS, 4MSOB GLS, benzyl GLS and I3M GLS) *in vitro* after incubation of 0.98 mM GLS with *Sinapis alba* myrosinase alone (Myr) or both myrosinase and 25 µg of **(A)** BoNSP2 (Myr + BoNSP2) or **(B)** BoNSP11 (Myr + BoNSP11). NSP activity assessed as the corresponding fold increase in nitrile formation from the five pure GLS standards *in vitro* under the same reaction conditions and in the presence of **(C)** BoNSP2 or **(D)** BoNSP11 compared to control assays with all reaction components except BoNSP. Values shown are the mean ± SD of three independent expression experiments (n = 3). The asterisk (*) denotes a statistically significant increase in nitrile formation (in µmol/g DW) from the corresponding GLS in the assay with BoNSP compared to the control assay without BoNSP for each GLS as determined by Kruskal−Wallis-ANOVA and Dunn’s *post-hoc* test or one-way ANOVA followed by Tukey’s *post-hoc* test (*p≤*0.05) **(A, B)**. The lowercase letters indicate a significant difference in the fold increase in nitrile formation from the five GLSs as determined by **(C)** Kruskal−Wallis-ANOVA and Dunn’s *post-hoc* test (H = 19.397, *p*<0.001) and **(D)** one-way ANOVA followed by Tukey’s *post-hoc* test (*p≤*0.05). DW – dry weight, 4MTB-CN – 5-(methylthio)pentanenitrile and 4MSOB-CN – 5-(methylsulfinyl)pentanenitrile.

Next, we compared the activity of BoNSP2 and BoNSP11 upon myrosinase-catalyzed hydrolysis of allyl GLS, 4MTB GLS, 4MSOB GLS, benzyl GLS and I3M GLS *in vitro.* In comparison to the control reactions without NSPs, BoNSP2 and BoNSP11 significantly increased CN formation from all five GLSs (**Figures 4A, B**; **Supplementary Table S6**), with increases ranging from 2-fold to 7.1-fold (**Figures 4C, D**; **Supplementary Table S6**). The effect of BoNSP2 on CN formation from allyl GLS, 4MTB GLS, 4MSOB GLS and benzyl GLS did not differ significantly, while it was slightly higher for I3M GLS compared to 4MSOB GLS (**Figure 4C**). The strongest increase in CN formation by BoNSP2 was observed for I3M GLS where the corresponding IACN (indole-3-acetonitrile) increased 7.1-fold (**Figure 4C**; **Supplementary Table S6**). Regarding BoNSP11, the increase of CN formation was similar upon hydrolysis of allyl GLS, 4MSOB GLS, benzyl GLS and I3M GLS, but significantly lower upon 4MSOB GLS hydrolysis compared to 4MTB GLS (**Figure 4D**).

### 3.5 BoNSP2 and BoNSP11 have similar optimal pH between pH 7 and pH 8

**Figure 5:**
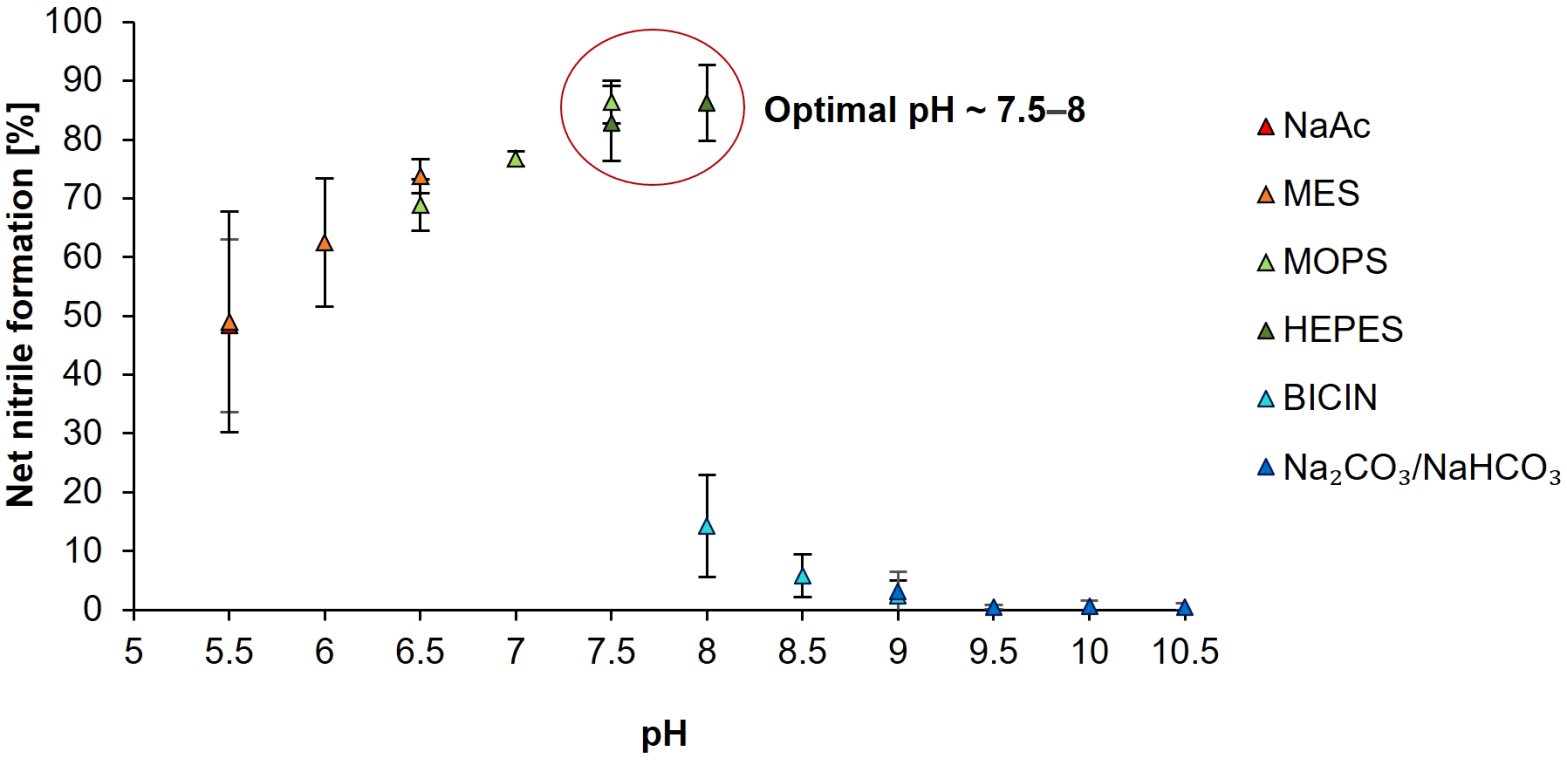
Influence of pH on the activity of purified BoNSP2 (XP_013609641.1). NSP activity was assessed as the proportion of allyl-CN [%] formed from allyl GLS in assays performed with NaAc (pH 5.5), MES (pH 5.5, 6 and 6.5), MOPS (pH 6.5, 7 and 7.5), HEPES (pH 7.5 and 8), BICINE (pH 8, 8.5 and 9) and Na_2_CO_3_/NaHCO_3_ (pH 9, 9.5, 10 and 10.5); in the presence of Fe^2+^ (34.3 µM) and 0.98 mM allyl GLS and myrosinase. Net nitrile formation [%] was calculated by subtracting the proportion of allyl-CN formed in the control reactions (Myr) from the proportion of nitriles formed in assays with myrosinase and BoNSP2 (Myr + BoNSP2) (**Supplementary Table S7**). Values shown are the mean ± SD of three independent expression experiments (n = 3).

The optimal pH for BoNSP2 activity was assessed in the pH range 5.5 to pH 10.5 using NaAc solution and five biological buffers, MES, MOPS, HEPES, BICINE and Na_2_CO_3_/NaHCO_3_, within their effective pH ranges. BoNSP2 activity assessed as the net CN formation (%) increased with increasing pH value till pH 8 (**Figure 5**) and activity was optimal at pH 7.5 to pH 8 (HEPES buffer) with net CN formation of 86% for allyl-CN (**Supplementary Table S7**). While buffer compounds used for the pH range from 5.5 to pH 8 did not seem to affect BoNSP activity, this was not the case for buffer compounds used for higher pH values. In assays with HEPES buffer at pH 8, the net CN formation rate was 86% whereas it was only 14% with BICINE buffer (**Figure 5**, **Supplementary Table S7**). Barely any BoNSP2 activity was detected from pH 9 to pH 10.5 with the Na_2_CO_3_/NaHCO_3_ buffer (**Figure 5**).

Further tests assessed the sensitivity of BoNSP2 and BoNSP11 to changes in pH using NaAc beyond its effective pH range (pH 4 to pH 12 for BoNSP2 and pH 4 to pH 9 for BoNSP11). Similar to the NSP assays with the six buffers with overlapping pH ranges (**Figure 5**), BoNSP2 activity (assessed as the net CN formation) was highest at pH 8 (83%) (**Supplementary Figure S6B**; **Supplementary Table S8**). However, with NaAc, BoNSP2 activity did not drop between pH 8 and pH 9 but stayed at the same level until pH 11 (**Figure 5**; **Supplementary Figure S6B**). Similar to BoNSP2, the highest BoNSP11 activity was at pH 7 and above reaching 73% of net CN formation (**Supplementary Figure S6D**; **Supplementary Table S8**).

## 4 Discussion

GLS hydrolysis in *B. oleracea* vegetables often results in the release of ITCs with implications for growers and consumers. ITCs derived from GLS hydrolysis in *Brassica* biomass applied to soil are able to suppress different soil-borne pests, pathogens and diseases thereby enhancing soil health (Pavana Praneetha et al., 2025). Moreover, ITCs have potential application in crop protection via foliar application as demonstrated by the antimicrobial properties of 4MSOB-ITC (sulforaphane)-enriched extracts applied to broccoli leaves (He et al., 2024). The use of biofumigants is advantageous as it can contribute towards more sustainable agriculture (Pavana Praneetha et al., 2025). Further, ITCs, have numerous biomedical properties beneficial for human health, for example, sulforaphane which is a potent anticarcinogen (Asif Ali et al., 2023; Hoch et al., 2024). Given the potential of ITC-based biofumigants and human health-beneficial properties, ITC formation from GLS hydrolysis would be ideal. However, due to ESP activity and abundance and the presence of BoNSPs, CNs are also formed in *B. oleracea*, reducing the proportion of ITCs (Mbudu et al., 2025; Púčiková et al., 2025). The NSP families in *Brassica rapa* (BrNSPs) and *Brassica napus* (BnNSPs) were identified and analyzed *in silico.* Also, the induction of putative *BrNSP* genes by *Spodoptera littoralis* attack and differential expression of candidate *BnNSP* genes upon hormone treatment was reported, however, functional characterization of *Brassica* NSPs is lacking *(*Han et al., 2023*;*Zhai et al., 2024*)*. Apart from our previous study in which we detected peptides derived from putative BoNSPs in kohlrabi (Mbudu et al., 2025), a systematic study of the NSP family in *B. oleracea* was lacking. Moreover, the functional characterization of NSPs in *Brassica* crops such as *Brassica oleracea* was still missing.

In this study, sixteen putative BoNSP genes on seven different chromosomes encoding proteins with significant sequence similarity to AtNSPs were identified (**Table 1**). All sixteen candidate sequences were designated as putative BoNSPs even if the encoding gene was presumably incomplete or incorrect (**Table 1**). This is meant to ensure consistency in the naming of the BoNSPs based on their genomic positions, should future research correct the gene models for BoNSP8 (presumably derived from an incorrect gene model), and BoNSP10, BoNSP13 and BoNSP14 (presumably incomplete proteins) (**Table 1**). Also, BoNSP10 and BoNSP13, which were presumed incomplete in this study based on the significantly shorter length of their Kelch domain regions, may be functional in other *Brassica oleracea* varieties. This would be in line with a study where the myrosinase gene *TGG6* (At1g51490.1), that is likely non-functional in five *A. thaliana* ecotypes (Wang et al., 2009), has functional alleles in ten other *A. thaliana* ecotypes (Fu et al., 2016). The identification of sixteen putative BoNSP candidates is in line with recent reports for *B. rapa*, where fifteen NSP candidates, distributed throughout the genome, were identified (Han et al., 2023) and in contrast to *B. napus* where seventy-two putative NSPs were identified (Zhai et al., 2024). Similar to *B. rapa*, about three times the number of NSP candidates were identified in *B. oleracea*, compared to *A. thaliana.* This is expected as past studies comparing the genome microstructures in selected genomic regions of *B. oleracea*, *B. rapa* and *B. napus* in relation to *A. thaliana* support the triplication of these *Brassica* genomes (Town et al., 2006). However, following the recent report on BnNSP candidates (Zhai et al., 2024), the higher number of NSPs in the closely related *B. napus* compared to *B. oleracea* is likely due to the greater complexity of the *B. napus* genome, which arose due to the interspecific hybridization of *B. rapa* and *B. oleracea* and subsequent genome rearrangements (Gu et al., 2024). Further, the recent detection of five putative BoNSPs in mature kohlrabi (Mbudu et al., 2025) suggests that these proteins are of functional importance in kohlrabi but this would need further investigation.

Domain prediction for the thirteen putative BoNSPs for which complete sequences were available revealed that similar to AtNSPs and BrNSP candidates (Burow et al., 2009; Han et al., 2023), the BoNSP candidates either exist as a chimera of up to five *N-*terminal jacalin-like lectin domains (IPR001229) and the Kelch domain or consist of the Kelch domain only (**Figure 1A**). This explains the huge variation in length (322–1064 amino acids) and molecular weight (34.8 kDa−116.4 kDa). As specifier proteins, including NSPs, have been described to be functional in promoting CN formation although they lack a jacalin domain (Burow et al., 2009; Kissen and Bones, 2009), this enzyme activity is associated with the Kelch domain. According to the available specifier protein crystal structures, the Kelch domain forms a six blade β-propeller structure with a central active site (Gumz et al., 2015; Zhang et al., 2017). The active site harbors three conserved amino acids (EXXXDXXXH) which coordinate an iron ion that interacts with the thiolate sulfur of GLS aglucones while the aglucone sulfate and the side chain interact with less conserved amino acid residues of the active site (Backenköhler et al., 2018; Gumz et al., 2015; Zhang et al., 2017). Based on multiple sequence alignment of the amino acid sequence of TaTFP (Gumz et al., 2015; Kuchernig et al., 2011), the thirteen putative BoNSPs and the ancestral protein XP_013585314.1, the iron-binding triad (E266, D270 and H274 in TaTFP) is strictly conserved in the putative BoNSP sequences except for BoNSP1, BoNSP3 and BoNSP16, and XP_013585314.1 (**Supplementary Figure S2**). In BoNSP1, the iron-binding triad appears to be modified and/or incomplete, and functional characterization is needed to test if this protein works as an NSP, lost this activity and/or fulfills a different function. The modified iron-binding triad (EXXXHXXXH) present in BoNSP3 and BoNSP16 was observed also for AtNSP5. These three proteins grouped together in the phylogenetic tree (**Figure 1A**). This suggests that they are functionally related. The likely ancestor of the putative BoNSP family, XP_013585314.1, possessed a modified iron binding triad (EXXXSXXXH) identical to what was reported for the AtNSP’s ancestral sequence At3g07720.1 (Brandt et al., 2014). As suggested for At3g07720.1 the modification in XP_013585314.1 likely results in lower iron-binding ability than the putative BoNSPs (Backenköhler et al., 2018). The iron-binding triad EXXXDXXXH was also strictly conserved in BoNSP8, BoNSP10 and BoNSP13, not included in our main analyses, further supporting their potential role as BoNSPs.

The function of the jacalin domains is presently unknown. The jacalin domains are sugar binding motifs, and jacalin domain containing proteins have been implicated in plant defense responses, therefore, the gain of more jacalin domains in BoNSP4, BoNSP5, BoNSP6 and BoNSP12 may be an adaptation to gain novel function in response to environmental stressors (Esch and Schaffrath, 2017). Biochemical tests would have to be carried out to gain more information on the possible roles of *B. oleracea* jacalin domains in environmental stress response. Maybe they have important roles in the cellular and organismic context of NSP function. For example, it should be tested if they are important for NSP localization, regulation or stabilization of the proteins *in planta*.

Phylogenetic analysis of the Kelch domain of the BoNSPs generally confirmed previous results regarding specifier protein evolution (Burow et al., 2009; Han et al., 2023; Kuchernig et al., 2012). Accordingly, XP_013585314.1 represents the ancestral protein, from which NSP function was derived, in *B. oleracea*. The *A. thaliana* counterpart, At3g07720.1, did not have NSP activity *in vitro* (Burow et al., 2009). For XP_013585314.1, this still needs to be tested. Based on our analysis and taking previous analyses into account (Kuchernig et al., 2012), BoNSP3 and BoNSP16 likely belong to the oldest group of proteins with specifier protein activity, those of the AtNSP5 clade. These proteins possess the Kelch domain without jacalin domains which may indicate a conserved function as previously suggested (Han et al., 2023). Interestingly, the remaining BoNSPs grouped according to their number of jacalin domains although the phylogenetic tree was built on the Kelch domain only. This may indicate further adaptations in the Kelch domain in response to properties or functions associated with the jacalin domains. BoNSP2 and BoNSP11, characterized *in vitro*, as well as four additional, putative BoNSPs were in the same subclade as AtNSP1−AtNSP4, suggesting similar roles. However, putative BoNSP4, BoNSP5, BoNSP6 and BoNSP15 formed a separate subclade. In contrast to the other BoNSPs with a maximum of two jacalin domains, these proteins possess three to five jacalin domains (**Figure 1A**). According to past studies, the jacalin domains in *A. thaliana* NSPs are likely derived from AtMBPs and the two jacalin domains in AtNSP4 were likely obtained from different sources (Burow et al., 2009; Kuchernig et al., 2012). Our phylogenetic analysis of BoNSP jacalin domains (**Supplementary Figure S5**) indicated that the jacalin domains which are directly linked to the *N*-terminus of the Kelch domain region are closely related and share a common origin. AtNSP jacalin domains were present in the same cluster as were two BoMBP2 and AtMBP isoforms as well as many of the BoNSP jacalin domains not directly linked to the Kelch domain region. A well supported second cluster contained additional BoNSP jacalin domains which seem to be related to another BoMBP isoform and are likely derived from a different source (**Supplementary Figure S5**). Although the tree topology within the clusters was poorly resolved, our results are in agreement with a past study on AtNSPs which concluded that the first jacalin domain upstream of the Kelch domain region is conserved among the proteins while the second upstream jacalin domain has a different origin (Burow et al., 2009; Han et al., 2023; Kuchernig et al., 2012).

BoNSP2 (XP_013609641.1) detected in eight kohlrabi parts and BoNSP11 (XP_013587057.1) only detected in the kohlrabi root in the first experiment of our previous study (Mbudu et al., 2025) were characterized *in vitro*. We confirmed the activity of BoNSP2 and BoNSP11 *in vitro* and then tested for dependency on Fe^2+^ concentration. It has been shown experimentally that specifier proteins contain iron (Backenköhler et al., 2018) and the iron-binding residues have been validated by mutational analysis (Brandt et al., 2014; Gumz et al., 2015). The Fe^2+^ promote CN formation through the abstraction of thioglucosidic bond S atom (Wittstock and Burow, 2007). This study found the previously described iron-binding residues to be largely conserved in the putative BoNSPs (**Supplementary Figure S2**) suggesting that Fe^2+^ is an active-site bound cofactor of BoNSPs. This is further supported by the enhanced net CN formation rate in the presence of added Fe^2+^ (**Figures 3B, D**). Similar to the study with AtNSP1 (Burow et al., 2009), low levels of CNs were also formed in the control assays containing allyl GLS as substrate and devoid of Fe^2+^ supplementation (**Figures 3A, C**). Addition of EDTA to assays with 34.3 µM mM Fe^2+^ and allyl GLS significantly reduced BoNSP2 and BoNSP11 activity (**Figures 3B, D**). This is in agreement with a past report where EDTA decreased AtNSP1 activity in assays with 4MTB GLS and benzyl GLS, however, the same effect was not observed for allyl GLS and 4MSOB GLS (Burow et al., 2009).

The significant increase in CN formation from all five tested GLSs is indicative of the broad substrate specificity of BoNSP2 and BoNSP11 and is in agreement with the studies with AtNSPs (Burow et al., 2009; Kissen and Bones, 2009; Kong et al., 2012). The broad substrate specificity of BoNSP2 and BoNSP11 indicates that the active site is relatively large and open to accommodate GLS aglucones with various side chains as has been suggested for AtNSP3 based on molecular modelling and substrate docking (Backenköhler et al., 2018; Eisenschmidt-Bönn et al., 2019; Mocniak et al., 2020). Despite the broad range of accepted substrates, the results suggest that BoNSP2 and BoNSP11 likely have different substrate specificities towards indole GLS aglucones (**Figures 4C, D**), consistent with the AtNSPs (Kong et al., 2012). BoNSP2 seemed to have the highest activity in assays with I3M GLS compared to aliphatic GLSs and benzyl GLS (**Figure 4C**). This finding is in line with the *in silico* study (Román et al., 2020) where molecular docking studies were performed with the broccoli NSP 100% identical to BoNSP2 (XP_013609641.1) (**Table 1**) and the aglucones of 4MSOB GLS, 4-hydroxy-I3M GLS (4OHI3M GLS), I3M GLS, 3-(methylsulfinyl)propyl GLS (3MSOP GLS), and allyl GLS. The I3M GLS-derived aglucone exhibited relatively lower binding affinity energy and a higher affinity constant with the broccoli NSP than the aglucones from 4MSOB GLS and allyl GLS at four different pH values (pH 1, pH 3, pH 5 and pH 7), suggesting a more stable complex (Román et al., 2020) and a stronger preference for the aglucone from I3M as substrate.

When fresh *Brassica* vegetables are chopped, ETNs and CNs are often the main GLS-derived products and shifting GLS hydrolysis towards ITC formation is desirable for enhanced nutritional value (Hanschen and Schreiner, 2017; Mbudu et al., 2025; Witzel et al., 2019). Shifting the pH during processing of *Brassica* vegetables to acidic or slightly alkaline pH can greatly impact ITC formation (Hanschen et al., 2017). The results of this current study demonstrate the sensitivity of BoNSP2 and BoNSP11 towards changes in pH, with both proteins showing highest activity between pH 7 to pH 8 (**Figure 5** and **Supplementary Figures S6B, D**) suggesting that adjustment of the pH value especially to acidic conditions should help to increase ITC formation. From the molecular modelling of BoNSP2 interactions with GLS aglucones *in silico* (Román et al., 2020), pH determines the stability of the NSP-GLS aglucone complexes which may explain the effect on NSP activity.

Taken together this study describes the presence of sixteen genes encoding potential BoNSP isoforms, along with a gene encoding the likely ancestral protein of the BoNSP candidates, in the genome of *B. oleracea*. Both BoNSP2 and BoNSP11 are expressed *in planta* (Mbudu et al., 2025) and are shown here to possess NSP activity *in vitro*. This is the first demonstration of functional NSPs in the genus *Brassica*. The BoNSP Kelch domain phylogeny suggests possible adaptations of Kelch domain amino acid sequences in association with an increasing number of *N*-terminal jacalin domains. Identification of the conserved iron-binding residues supports the role of Fe^2+^ as a cofactor of BoNSPs. The precise biological and ecological functions of BoNSPs can be further explored in knockout or overexpression studies, and by studying BoNSP expression in different tissues and ontogenic stages, or upon hormone treatments (Zhai et al., 2024). This study paves the way for further studies focused on deciphering NSP function and ways to optimize ITC formation *in planta* for increased crop resilience and nutritional quality.

## Data Availability

The original contributions presented in the study are included in the article/**Supplementary Material**. Further inquiries can be directed to the corresponding author.
