# Supplementary material for: Biochemical characterization of two Brassica oleracea nitrile-specifier proteins
Source: Front Plant Sci. 2026 Jan 29;17:1740844. doi: 10.3389/fpls.2026.1740844 (PMC12894210; doi:10.3389/fpls.2026.1740844)
Supplement: Supplementary file 1 [file DataSheet1.docx]

Supplementary Material


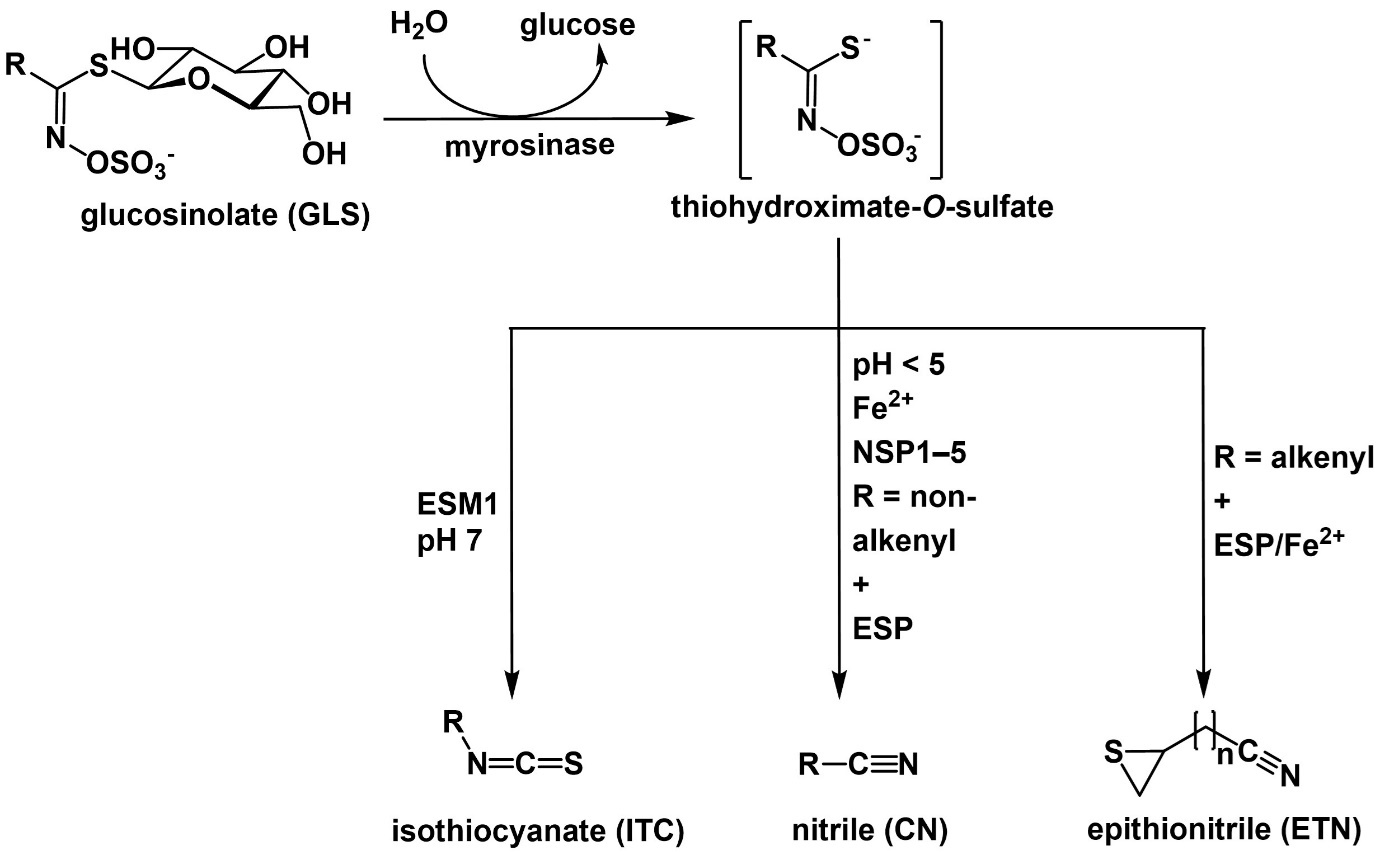


**Figure S1** Schematic representation of the enzymatic hydrolysis of glucosinolates (GLSs) by myrosinase and the hydrolysis products formed under different reaction conditions. All GLSs possess the same core structure but vary in their side chain (R). The unstable intermediate (thiohydroximate-O-sulfate) can rearrange spontaneously to form ITCs, however, GLS hydrolysis can be diverted towards CN and ETN formation under certain reaction conditions. CN formation is enhanced in the presence of NSPs, at acidic pH (<5) and in the presence of ferrous ions. When the GLS side chain has a terminal unsaturation, ESP and ferrous ions promote ETN formation (Wittstock & Burow, 2010).

**
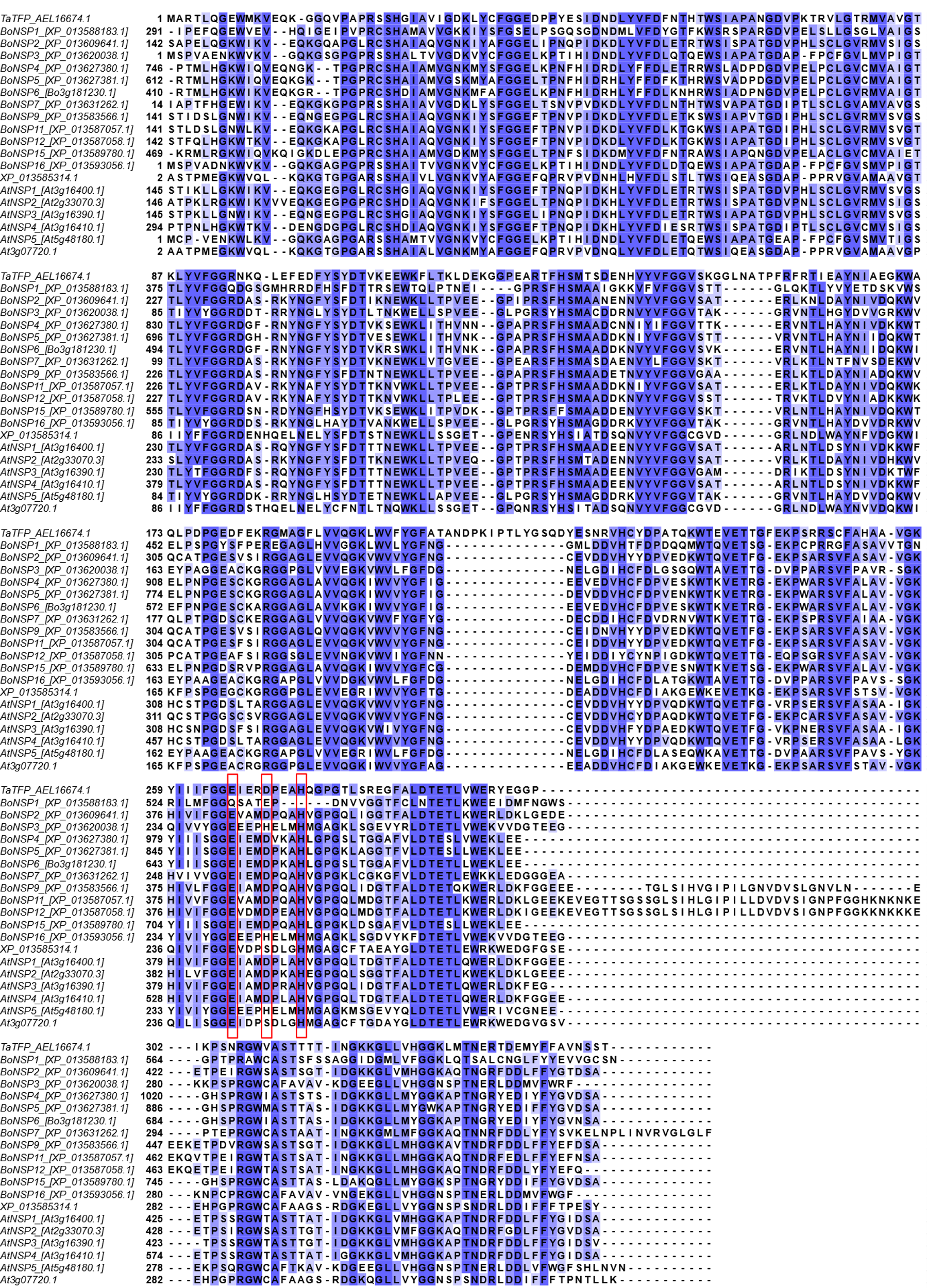
**

**Figure S2** Multiple sequence alignment of the Kelch domains of the *Thlaspi arvense* thiocyanate forming protein (TaTFP) (Kuchernig *et al*., 2011; Gumz *et al*., 2015), twelve putative BoNSPs (BoNSP1–BoNSP7, BoNSP9, BoNSP11, BoNSP12, BoNSP15 and BoNSP16), the likely ancestor of the BoNSPs, XP_013585314.1, the five AtNSPs (AtNSP1–AtNSP5) and the ancestral protein of the AtNSPs, At3g07720.1. The conserved iron binding residues (E266, D270 and H274 in TaTFP) are highlighted by the red boxes. The sequences were aligned using ClustalW in Clustal Omega (Madeira *et al*., 2024) and visualized in Jalview version 2.11.4.1 (Waterhouse *et al*., 2009). The percent identity colour scheme was selected in Jalview and the increase in colour intensity indicates increased sequence conservation from 41 % to 100 %.


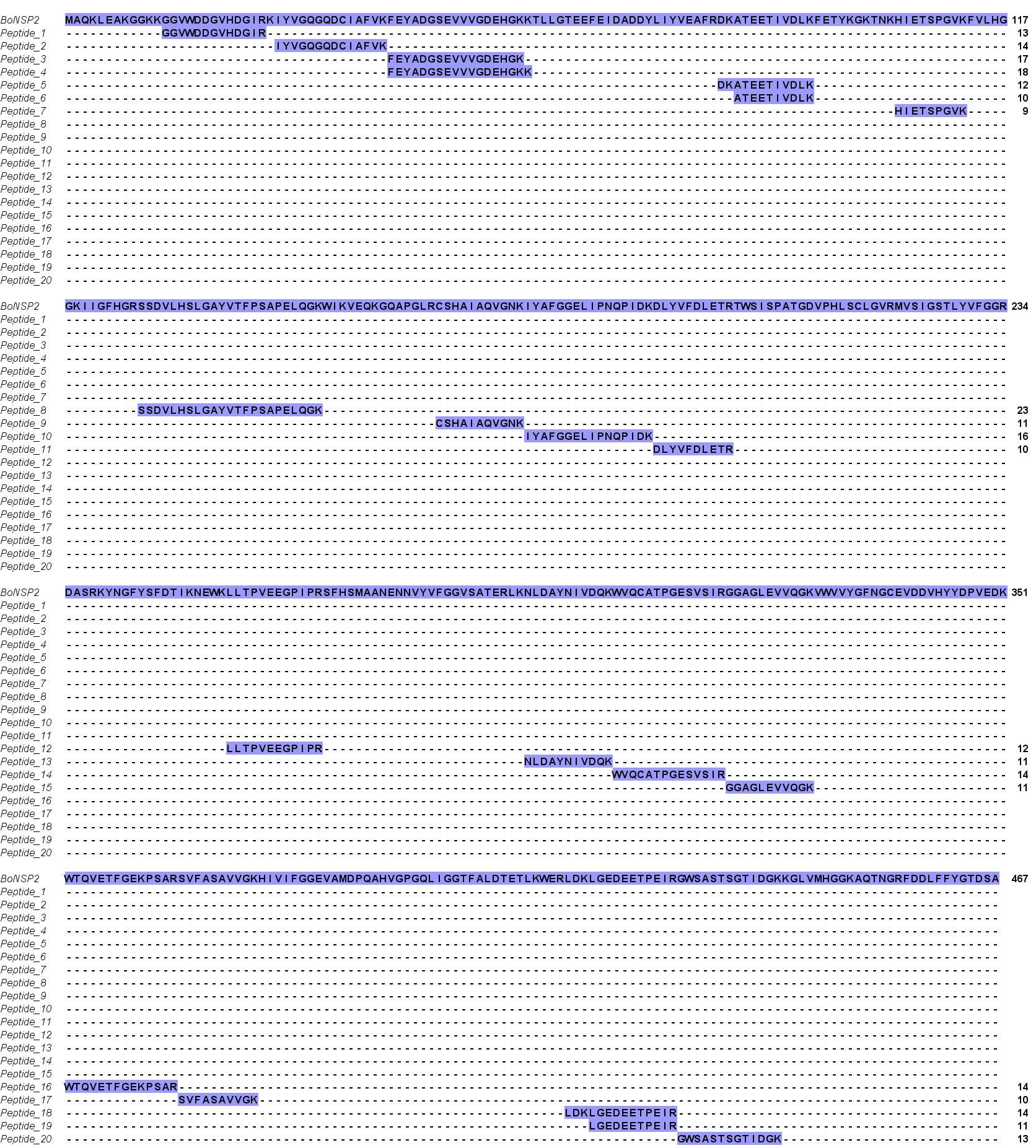


**Figure S3** Alignment of the amino acid sequences of the twenty peptides (peptides 1 to peptide 20) assigned to BoNSP2 (XP_013609641.1) in the mature kohlrabi proteome in our recent study (Mbudu et al., 2025) and also cloned, expressed and characterized in this current study. The peptides were aligned to the protein sequences by ClustalW in Clustal Omega (Madeira et al., 2024) and visualized in Jalview version 2.11.4.1 (Waterhouse et al., 2009). The BLOSUM62 colour scheme was selected and applied in Jalview. The dark blue colour indicates that a residue matches the consensus sequence at that specific position.


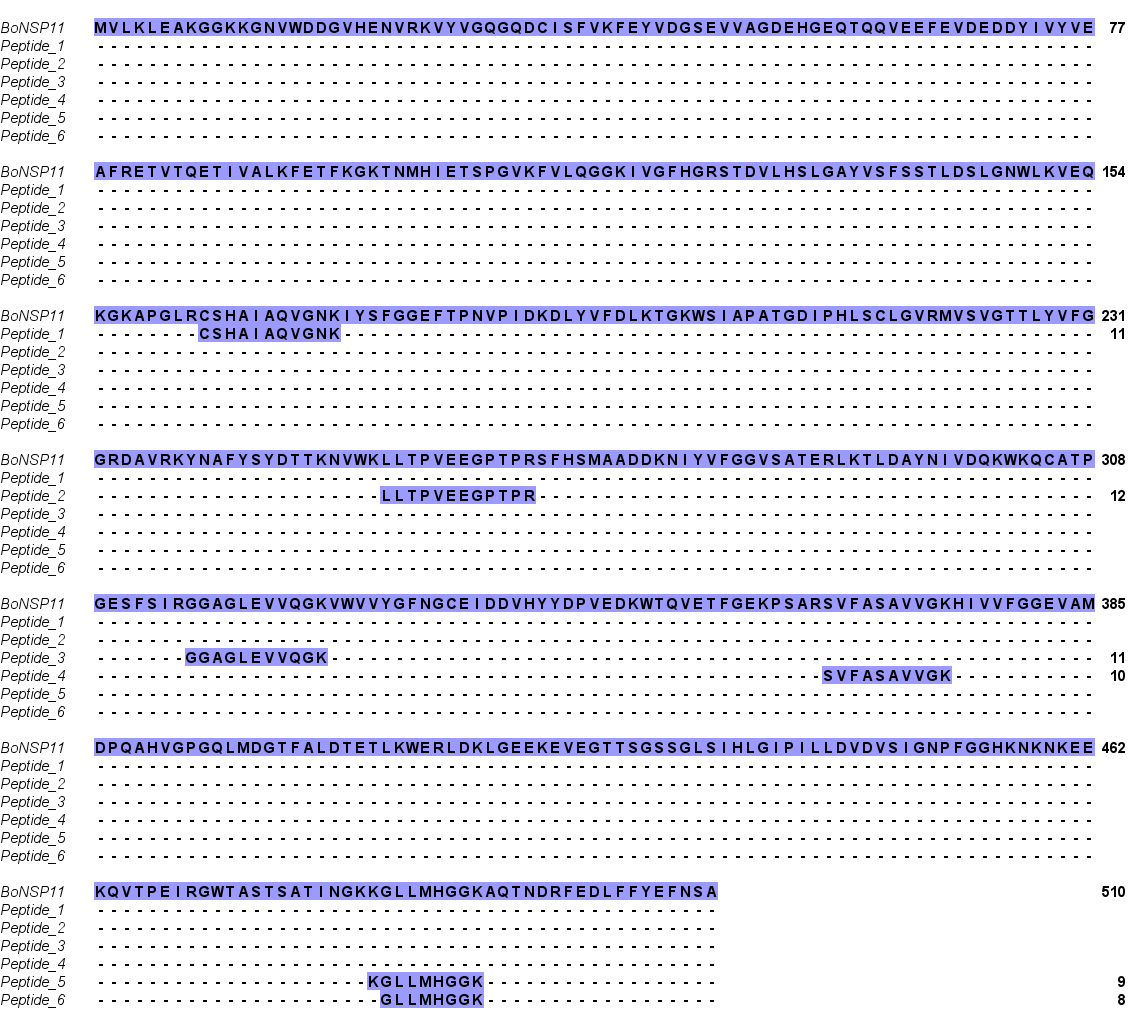


**Figure S4** Alignment of the amino acid sequences of the six peptides (peptides 1 to peptide 6) assigned to BoNSP11 (XP_013587057.1) in the mature kohlrabi proteome in our recent study (Mbudu et al., 2025) and also cloned, expressed and characterized in this current study. The peptides were aligned to the protein sequences by ClustalW in Clustal Omega (Madeira et al., 2024) and visualized in Jalview version 2.11.4.1 (Waterhouse et al., 2009). The BLOSUM62 colour scheme was selected and applied in Jalview. The dark blue colour indicates that a residue matches the consensus sequence at that specific position.


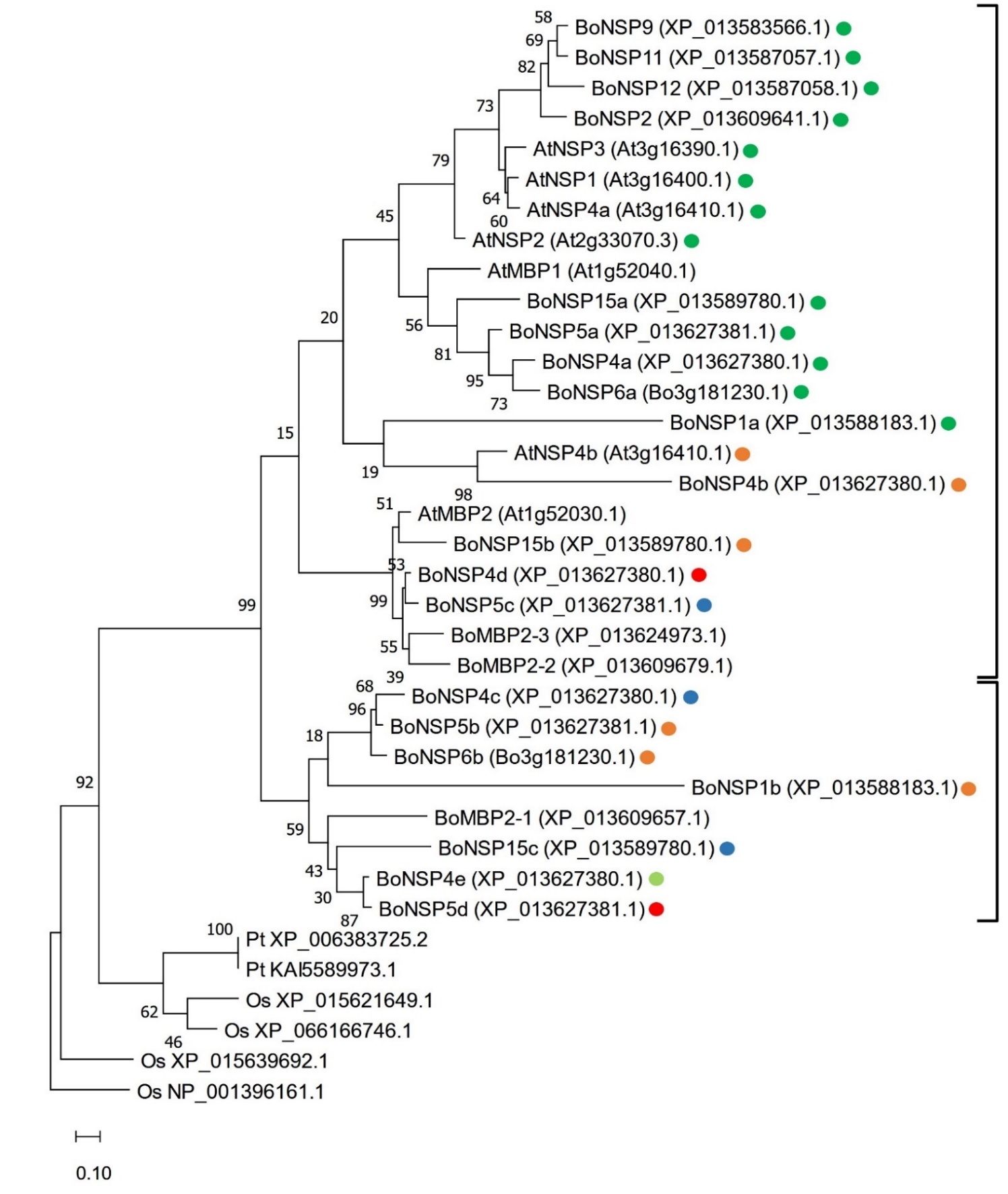


**Figure S5** Phylogenetic analysis of the putative *B. oleracea* jacalin domains based on their amino acid sequences. Twenty jacalin domains sequences from nine putative BoNSPs (BoNSP1, BoNSP2, BoNSP4–BoNSP6, BoNSP9, BoNSP11, BoNSP12 and BoNSP15) and five jacalin domains from AtNSP1–4, and the full-length amino acid sequences of three putative BoMBP2 isoforms (XP_013609657.1, XP_013609679.1 and XP_013624973.1), three BoMBP2 orthologues in rice (*Oryza sativa Japonica Group*, Os) and two in poplar (*Populus trichocarpa*, Pt), AtMBP1 (At1g52040.1), AtMBP2 (At1g52030.1) and *Oryza sativa* NP_001396161.1 as outgroup were aligned in MEGA11. The evolutionary history was inferred using the Maximum Likelihood method and Whelan and Goldman model (Whelan, S. and Goldman, N. (2001)) in MEGA11 (Tamura K., Stecher G., and Kumar S. (2021)). The tree with the highest log likelihood (-5571.59) is shown. The bootstrap values are shown next to the branches. The tree is drawn to scale and the branch lengths correspond to the number of substitutions per site.


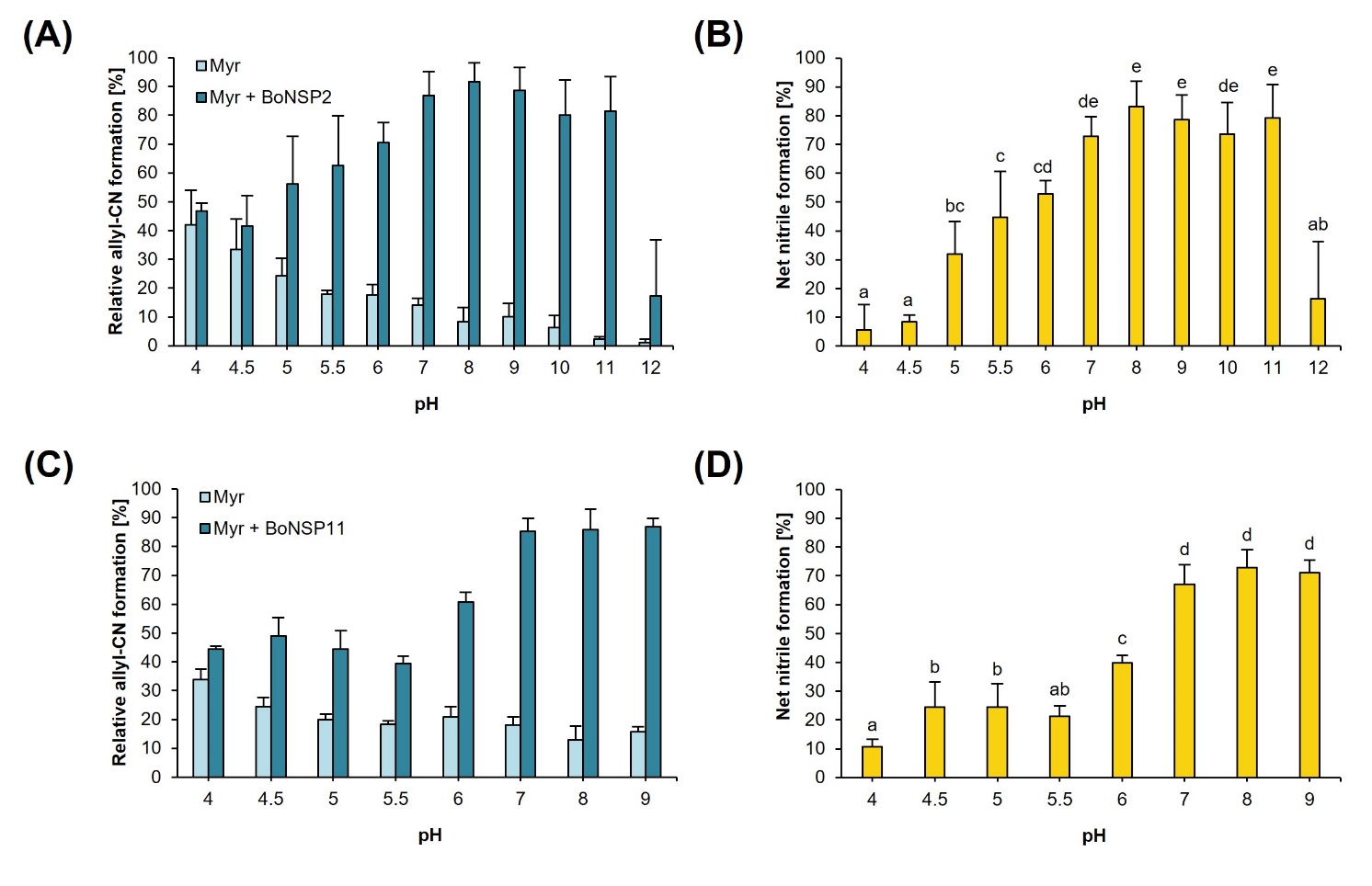


**Figure S6** Influence of pH on the activity of the purified BoNSP2 and BoNSP11. NSP activity was assessed as the proportion of allyl-CN [%] formed from allyl GLS. BoNSP2 activity was measured in assays performed with (A) NaAc set from pH 4 to pH 12, 34.3 µM Fe^2+^ and 1.18 mM allyl GLS. The assay for BoNSP11 was conducted with (C) NaAc set from pH 4 to pH 9. The net nitrile formation [%] for (B) BoNSP2 and (D) BoNSP11 was calculated by subtracting the proportion of allyl-CN formed in the control reactions (background nitrile formation) with *Sinapis alba* myrosinase alone (Myr) from the proportion of nitriles formed in assays with myrosinase and BoNSP2 (Myr + BoNSP2) or BoNSP11 (Myr + BoNSP11). Values shown are the mean ± SD of three independent expression experiments (n = 3). The lowercase letters indicate a significant difference in net nitrile formation in the presence of (B) BoNSP2 and (D) BoNSP11 at different pH values as determined by one-way ANOVA followed by Tukey’s post-hoc test (p≤0.05).
